# Systemic longitudinal immune profiling identifies proliferating Treg cells as predictors of immunotherapy benefit: biomarker analysis from the phase 3 CONTINUUM and DIPPER trials

**DOI:** 10.1038/s41392-024-01988-w

**Published:** 2024-10-23

**Authors:** Sai-Wei Huang, Wei Jiang, Sha Xu, Yuan Zhang, Juan Du, Ya-Qin Wang, Kun-Yu Yang, Ning Zhang, Fang Liu, Guo-Rong Zou, Feng Jin, Hai-Jun Wu, Yang-Ying Zhou, Xiao-Dong Zhu, Nian-Yong Chen, Cheng Xu, Han Qiao, Na Liu, Ying Sun, Jun Ma, Ye-Lin Liang, Xu Liu

**Affiliations:** 1 Department of Radiation Oncology, State Key Laboratory of Oncology in South China, Collaborative Innovation Center of Cancer Medicine, Guangdong Key Laboratory of Nasopharyngeal Carcinoma Diagnosis and Therapy, Guangdong Provincial Clinical Research Center for Cancer, Sun Yat-sen University Cancer Center, Guangzhou, PR China; 2 Department of Experimental Research, State Key Laboratory of Oncology in South China, Collaborative Innovation Center of Cancer Medicine, Guangdong Key Laboratory of Nasopharyngeal Carcinoma Diagnosis and Therapy, Guangdong Provincial Clinical Research Center for Cancer, Sun Yat-sen University Cancer Center, Guangzhou, PR China; 3 Cancer Center, Union Hospital, Tongji Medical College, Huazhong University of Science and Technology, Wuhan, PR China; 4 Department of Radiation Oncology, The First People’s Hospital of Foshan, Foshan, PR China; 5 Department of Pathology, The First People’s Hospital of Foshan, Foshan, PR China; 6 Department of Oncology, Panyu Central Hospital, Guangzhou, PR China; 7 Department of Oncology, The Affiliated Cancer Hospital of Guizhou Medical University, Guiyang, PR China; 8 Department of Oncology, Xiangya Hospital, Central South University, Changsha, PR China; 9 Department of Radiation Oncology, Affiliated Tumor Hospital of Guangxi Medical University, Nanning, PR China; 10 Department of Radiation Oncology, Cancer Center, West China Hospital, Sichuan University, Chengdu, PR China

**Keywords:** Predictive markers, Immunotherapy, Head and neck cancer, Lymphocytes

## Abstract

The identification of predictors for immunotherapy is often hampered by the absence of control groups in many studies, making it difficult to distinguish between prognostic and predictive biomarkers. This study presents biomarker analyses from the phase 3 CONTINUUM trial (NCT03700476), the first to show that adding anti-PD-1 (aPD1) to chemoradiotherapy (CRT) improves event-free survival (EFS) in patients with locoregionally advanced nasopharyngeal carcinoma. A dynamic single-cell atlas was profiled using mass cytometry on peripheral blood mononuclear cell samples from 12 pairs of matched relapsing and non-relapsing patients in the aPD1-CRT arm. Using a supervised representation learning algorithm, we identified a Ki67^+^ proliferating regulatory T cells (Tregs) population expressing high levels of activated and immunosuppressive molecules including FOXP3, CD38, HLA-DR, CD39, and PD-1, whose abundance correlated with treatment outcome. The frequency of these Ki67^+^ Tregs was significantly higher at baseline and increased during treatment in patients who relapsed compared to non-relapsers. Further validation through flow cytometry (*n* = 120) confirmed the predictive value of this Treg subset. Multiplex immunohistochemistry (*n* = 249) demonstrated that Ki67^+^ Tregs in tumors could predict immunotherapy benefit, with aPD1 improving EFS only in patients with low baseline levels of Ki67^+^ Tregs. These findings were further validated in the multicenter phase 3 DIPPER trial (*n* = 262, NCT03427827) and the phase 3 OAK trial of anti-PD-L1 immunotherapy in NSCLC, underscoring the predictive value of Ki67^+^ Treg frequency in identifying the beneficiaries of immunotherapy and potentially guiding personalized treatment strategies.

## Introduction

The advent of immune checkpoint inhibitors (ICI) has transformed cancer treatment over the past decade. However, the limited response rate of 20–30% among patients with solid tumors, coupled with the potential for immunotherapy-related toxicities and the relatively high cost, underscores the critical need for optimal patient selection. In this context, numerous biomarkers for ICI treatment have been reported. However, only a few of them have been validated in a randomized setting and implemented in clinical practice, such as programmed cell death 1 ligand 1(PD-L1) expression, tumor mutation burden (TMB), and microsatellite instability.^1,2^ The predictive value of these biomarkers is not consistent across different types of cancer, indicating that other biological factors determining the efficacy of ICI remain undiscovered. For example, while PD-L1 expression has been considered a predictive biomarker in several cancers, recent studies have shown that patients with nasopharyngeal carcinoma (NPC) can benefit from anti-PD-1 immunotherapy regardless of PD-L1 expression.^3–5^ Additionally, few genetic mutations are identified in NPC, indicating that TMB is not a useful predictor in this setting.^6^ These results highlight the unmet need for novel predictive markers for immunotherapy.

Peripheral blood mononuclear cell (PBMC) samples are ideal sources for biomarker development due to their minimally invasive nature and clinical feasibility for dynamically monitoring tumor evolution and therapy responses. Although the immune cell composition in peripheral blood is different from that in the tumor microenvironment (TME), previous studies have shown that not only the immune subsets identified in the periphery could be detected in the TME, but also the expression of some molecules on the immune subsets in the tumors correlated with that in matched blood,^7,8^ making it possible to use PBMC samples for biomarker discovery. High-dimensional mass cytometry, or CyTOF, is a single-cell analysis technique used in biomarker studies that can measure over 40 cellular markers simultaneously with higher sensitivity and lower detection overlap and background staining compared to conventional flow cytometry.^9^ To cope with the high dimensionality of CyTOF data, several algorithms have been developed to facilitate cell clustering and annotation as well as biomarker discovery, which render CyTOF powerful for immunophenotyping to identify prognostic or predictive immune biomarkers in cancer clinical trials where samples are rare and limited.

The phase 3 CONTINUUM trial was the first to show that adding anti-programmed cell death 1 (aPD1) treatment to chemoradiotherapy (CRT) improves event-free survival (EFS) in locoregionally advanced NPC.^10^ Leveraging the benefits of a randomized setting, the CONTINUUM trial ensured rigorous sample collection, providing a high-quality dataset that included sequential PBMC samples and baseline primary tumor tissue samples for comprehensive biomarker analysis. In this study, we performed immune profiling using CyTOF on longitudinal PBMC samples and identified the Ki67^+^ Treg subset as strongly associated with relapse. This subset’s predictive value for immunotherapy benefits was further confirmed through flow cytometry and multiplex immunohistochemistry (mIHC). Moreover, we confirmed the predictive value of Ki67^+^ Tregs in an independent phase 3 trial (DIPPER) for NPC. Additional studies in NSCLC and melanoma cohorts—both randomized and single-arm—further demonstrated the robustness and general applicability of our findings.

## Results

### Study design and patient characteristics

**Fig. 1:**
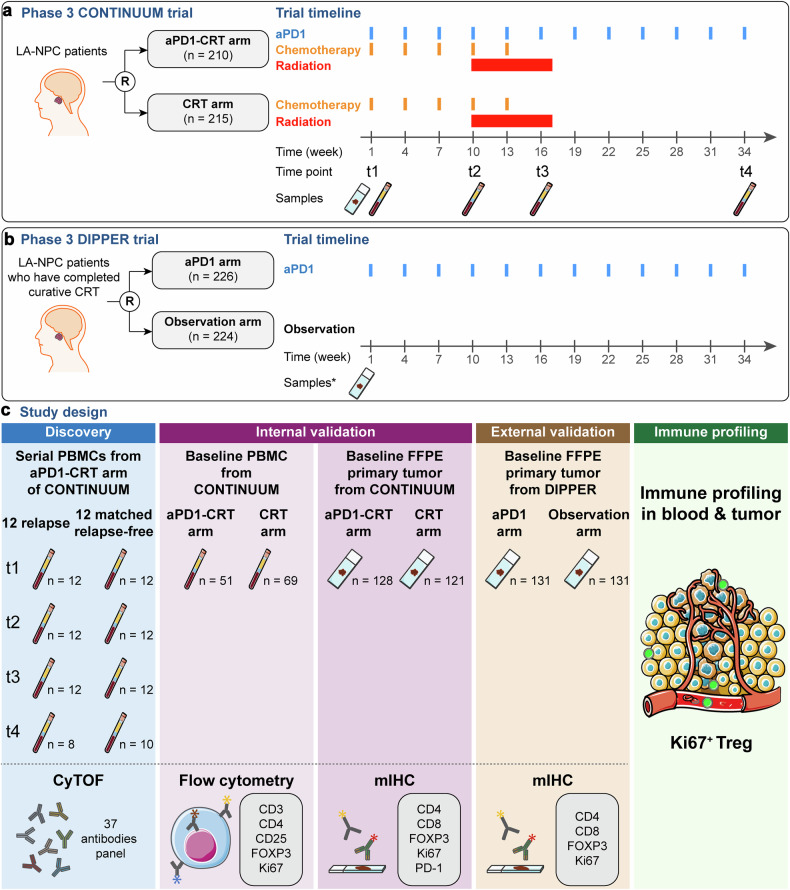
Study design. Overview of trial design and sample collection in the CONTINUUM trial (**a**) and DIPPER trial (**b**). In this study, CRT refers to standard therapy for LA-NPC patients that comprises induction chemotherapy and concurrent chemoradiotherapy. *Archived tumor samples were collected before CRT in DIPPER trial. **c** Overview of the study design. R randomization, LA-NPC locoregionally advanced nasopharyngeal carcinoma, CyTOF mass cytometry, mIHC multiplex immunohistochemistry, PBMC peripheral blood mononuclear cell, FFPE formalin-fixed paraffin-embedded. Parts of the figure were drawn using elements from Servier Medical Art (https://smart.servier.com/), under CC BY 4.0

In the phase 3 CONTINUUM trial^10^ (Fig. 1a), a total of 425 patients were randomized to receive either anti-PD-1 plus chemoradiation (aPD1-CRT arm, *n* = 210) or chemoradiation alone (CRT arm, *n* = 215). The primary endpoint was EFS, and the median follow-up period was 41.9 months (interquartile range (IQR) 38.0–44.8). Of the 389 patients alive at the data cut-off date, 366 (94%) had a follow-up of more than 36 months, and the last patient enrolled had a follow-up of 35.0 months. The trial prospectively designed biomarker analyses and collected longitudinal blood samples and baseline primary tumor samples (Fig. 1a). Among the 29 patients in the aPD1-CRT arm who developed disease relapse, 12 patients had longitudinal PBMC samples available at three or more time points throughout the treatment (Supplementary Fig. 1). As our primary goal was to identify biomarkers for PD-1 blockade through the analysis of longitudinal peripheral immune profiles throughout treatment, we included PBMC samples for CyTOF analysis from all these 12 patients who were matched 1:1 with relapse-free patients in the aPD1-CRT arm, aiming to balance age, sex, stage, and PD-L1 expression (Supplementary Table 1). Subsequently, the findings were validated in all remaining patients with available baseline PBMC samples (total *n* = 120; aPD1-CRT, *n* = 51; CRT, *n* = 69), and in all patients with available baseline primary tumor samples (total *n* = 249; aPD1-CRT, *n* = 128; CRT, *n* = 121).

In the phase 3 DIPPER trial^11^ (Fig. 1b), a total of 450 patients who completed curative chemoradiation were randomized to receive either anti-PD-1 (aPD1 arm, *n* = 226) or observation (Observation arm, *n* = 224). The primary endpoint was EFS, and the median follow-up time was 39 months (IQR 33–50). Overview of the study design has been summarized in Fig. 1c. In the DIPPER trial validation cohort, all available pretreatment FFPE primary tumor samples (*n* = 262) underwent mIHC staining. Demographic and baseline characteristics in patients included in the current biomarker study were similar to those in the intention-to-treat population for both trial cohorts (Supplementary Tables 2 and 3). Baseline patient characteristics were well balanced between the aPD1-treated arm and the control arm in both cohorts (Supplementary Tables 4 and 5).

### Different patterns of immune cell populations before and during aPD1 treatment

We utilized CyTOF to conduct high-dimensional single-cell analysis of PBMC using a panel of 37 canonical immune cell markers (Supplementary Table 6). This analysis identified a total of 10,487,969 live CD45^+^ immune cells from the 90 PBMC samples of the 12 pairs of matched patients (Supplementary Fig. 2). Unsupervised clustering via FlowSOM^12^ revealed six clusters comprising major populations in peripheral blood, including CD4^+^ T cells, CD8^+^ T cells, CD19^+^ B cells, CD33^+^ myeloid cells, and CD56^+^ natural killer (NK) cells (Supplementary Fig. 3a). Feature plots demonstrated robust expression of canonical markers within the anticipated immune subsets (Supplementary Fig. 3b, c). Myeloid cells were found to be the most abundant cells in PBMCs, accounting for 28.4% of total PBMCs, while the proportions of CD8^+^ T cells and CD4^+^ T cells were comparable at 21.7% and 21.6%, respectively. B cells constituted a relatively small portion at 4.4% (Supplementary Fig. 3c). Importantly, the phenotypic composition of these cell types varied among individual patients, indicating substantial immune heterogeneity among individuals. For example, patient A08 displayed a large proportion of CD8^+^ T cells and a small proportion of myeloid cells in PBMCs, whereas patient B10 exhibited the opposite pattern (Supplementary Fig. 3d, e).

The patterns of cell composition exhibited changes during treatment (Supplementary Fig. 3f). CD8^+^ T cells showed minimal change during aPD1 and induction chemotherapy (aPD1-IC) (t1–t2), consistent with our previous study on the immune modulation effects of IC.^13^ However, during aPD1 and concurrent chemoradiotherapy (aPD1-CCRT) (t2–t3), CD8^+^ T cells experienced a dramatic decrease, possibly due to the relatively higher radiation sensitivity of lymphocytes compared to myeloid cells.^14^ Subsequently, during aPD1 alone adjuvant therapy (t3–t4), the level of CD8^+^ T cells increased but did not recover to the baseline level. The changes in CD4^+^ T cells exhibited a pattern similar to that of CD8^+^ T cells, except for a further decrease during aPD1 alone adjuvant therapy (t3–t4). The circulating B cell level exhibited a moderate reduction during the aPD1-IC and aPD1-CCRT period (t1–t3), but returned to the baseline level at the end of aPD1 alone adjuvant therapy (t4). The frequency of myeloid cells increased during the aPD1-IC and aPD1-CCRT period (t1–t3) and decreased during aPD1 alone adjuvant therapy (t3–t4). These dynamic changes in immune cell composition throughout the treatment period provide valuable insights into the effects of different therapeutic interventions on the immune system.

The immune cell frequencies were compared between the 12 pairs of matched patients who developed posttreatment relapse and those who remained relapse-free. Overall, the dynamic changes of various cell types in the two groups were quite consistent. We observed no significant differences in major immune cell frequencies, except for a higher frequency of B cells in the relapse group at the end of aPD1 alone adjuvant therapy (t4, *P* = 0.021; Supplementary Fig. 3g). This finding prompted us to further study subpopulations within each major cell type.

### ICI failure correlates with high levels of Treg and naïve cells as well as low levels of terminal effector cells

**Fig. 2:**
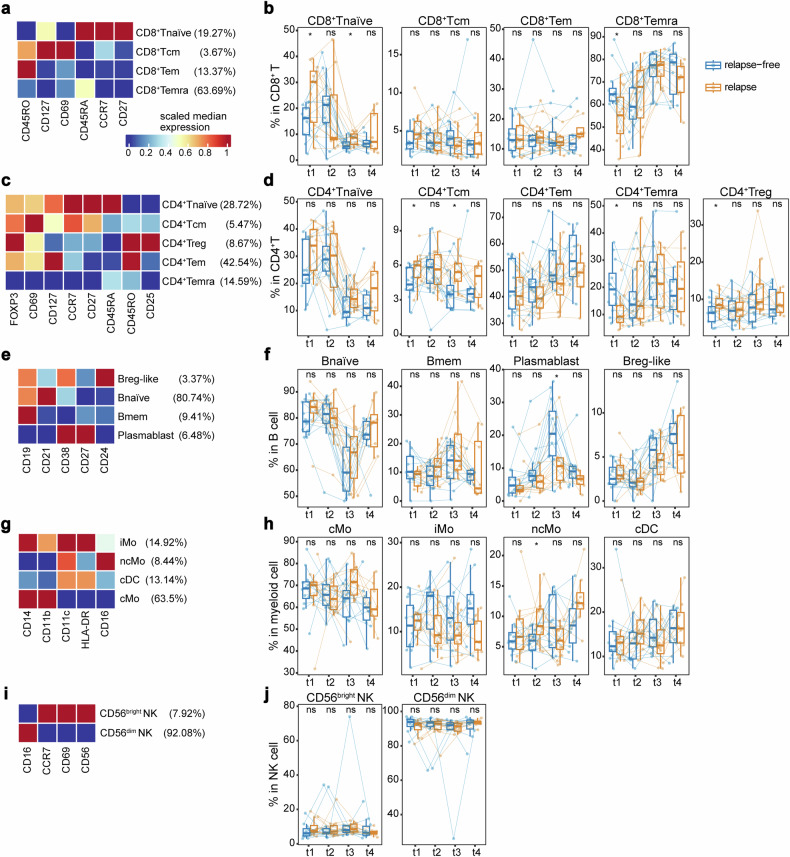
ICI failure correlates with high levels of Treg and naïve cells as well as low levels of terminal effector cells. Heatmaps showing expression of canonical markers and phenotypic composition of each subpopulation of CD8^+^ T cells (**a**), CD4^+^ T cells (**c**), CD19^+^ B cells (**e**), CD33^+^ myeloid cells (**g**), and NK cells (**i**), respectively. Box plots showing frequencies of each subpopulation of CD8^+^ T cells (**b**), CD4^+^ T cells (**d**), CD19^+^ B cells (**f**), CD33^+^ myeloid cells (**h**), and NK cells (**j**), respectively, between the relapse-free group and the relapse group at each time point of blood collection. For comparisons at each time point, *n* = 24 for t1–t3 and *n* = 18 for t4. Box plots represent the median (center line), the IQR (box), and the farthest data point within a maximum of 1.5 × IQR (whiskers). All *P* values were calculated using two-sided Wilcoxon tests. *P < 0.05; ns not significant. Tnaïve naïve T cell, Tcm central memory T cell, Tem effector memory T cell, Temra terminally differentiated effector memory T cell, Treg regulatory T cell, Bnaïve naïve B cell, Bmem memory B cell, Breg regulatory B cell, cMo classical monocyte, iMo intermediate monocyte, ncMo non-classical monocyte, cDC conventional dendritic cell, NK natural killer cell

To gain insights into the differences in the subpopulations of immune cells between the relapse group and the relapse-free group, we initially conducted FlowSOM subclustering to examine CD8^+^ and CD4^+^ T cells, respectively. For CD8^+^ T cells, we identified CD45RA^+^CCR7^+^ naïve cells (CD8^+^Tnaïve), CD45RA^−^CCR7^+^ central memory cells (CD8^+^Tcm), CD45RA^−^CCR7^−^ effector memory cells (CD8^+^Tem), and CD45RA^+^CCR7^−^ terminally differentiated effector memory cells (CD8^+^Temra)^15,16^ (Fig. 2a). Patients who experienced relapse exhibited a significantly higher frequency of CD8^+^Tnaïve cells (*P* = 0.039) and a lower frequency of CD8^+^Temra cells (*P* = 0.014) compared to relapse-free patients at baseline (Fig. 2b). At the conclusion of aPD1-CCRT therapy (t3), patients who experienced relapse still demonstrated a higher frequency of circulating CD8^+^Tnaïve cells (*P* = 0.045; Fig. 2b). CD4^+^ T cells were subdivided using the same approach, with an additional CD25^+^CD127^−^ regulatory T cell subpopulation (CD4^+^Treg) identified (Fig. 2c). Patients who experienced relapse showed a significantly higher frequency of CD4^+^ Treg cells (*P* = 0.033) and a lower baseline level of CD4^+^ Temra cells (*P* = 0.014; Fig. 2d) compared to relapse-free patients. Taken together, higher baseline levels of Treg and naïve cells, as well as lower levels of terminal effector cells, are associated with an increased risk of relapse, indicating that patients who experienced relapse after anti-PD-1 therapy were in an immune hypo-activated state.

We also subdivided CD19^+^ B cells, myeloid cells, and NK cells into subpopulations based on canonical markers and examined their dynamic changes during treatment (“Materials and Methods”, Fig. 2e–j). Comparisons of cell frequencies between groups did not reveal any significant differences, except for a lower level of plasmablast at the conclusion of aPD1-CCRT therapy (t3) and a higher level of non-classical monocytes (ncMo) at the end of aPD1-IC therapy (t2) in the relapse group (*P* = 0.014 and 0.017, respectively; Fig. 2f, h).

### Baseline peripheral Ki67^+^ Treg cells predict disease relapse and efficacy of anti-PD-1 therapy

**Fig. 3:**
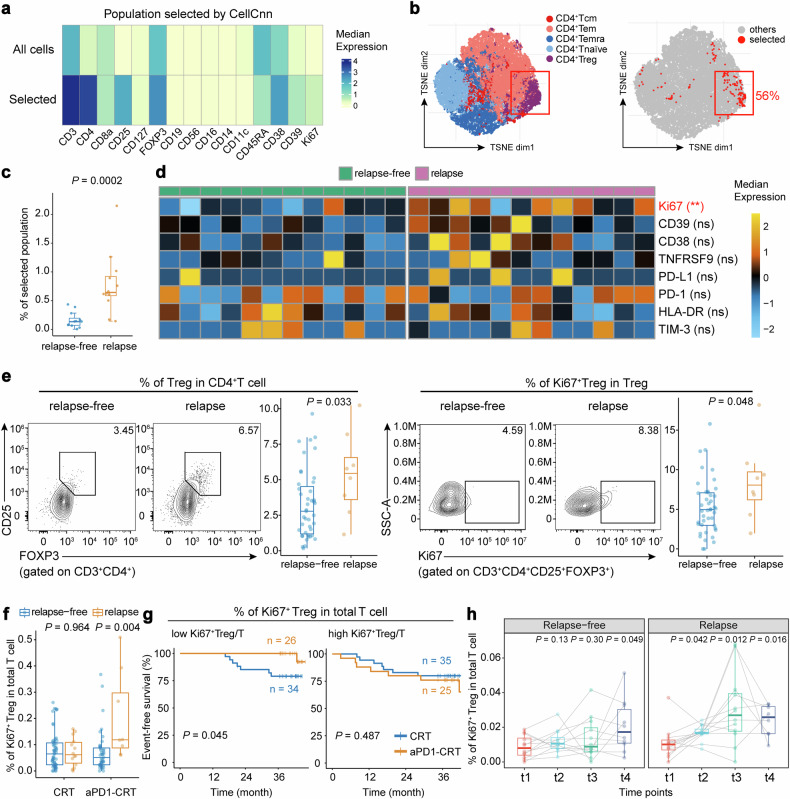
Peripheral Ki67^+^ Treg cells predict efficacy and disease relapse after anti-PD-1 therapy. **a** Heatmap showing phenotype of cells selected by CellCnn (*n* = 15,132). **b** T-SNE plots showing the five CD4^+^ T cell clusters we identified (left) and cells selected by CellCnn which were mainly located within the CD4^+^ Treg cluster (right). Red box indicates the CD4^+^ Treg cluster. **c** Box plot showing a higher frequency of cells discovered by CellCnn in patients from the relapse group than those from the relapse-free group. **d** Heatmaps showing comparisons of the median expression of functional markers in Treg cells from patients in the relapse-free group and the relapse group at baseline (*n* = 24). Bars at the top of the heatmaps represent individual samples from relapse-free group (green) and relapse group (pink). **P < 0.01, (marked in red); ns not significant. **e** Representative flow plots and box plots showing the frequency of Treg cells in CD4^+^ T cells (left) and Ki67^+^ Treg cells in Treg cells (right) between the relapse group and the relapse-free group compared in the aPD1-CRT arm (*n* = 51). **f** Box plots showing the frequency of Ki67^+^ Treg cells in total T cells between the relapse group and the relapse-free group compared in the CRT arm and in the aPD1-CRT arm, respectively (*n* = 69 and 51, respectively). *P* values were calculated by two-sided Wilcoxon tests in (**c**–**f**). **g** Kaplan–Meier curves comparing EFS improvements for anti-PD-1 plus CRT versus CRT alone in patients stratified by the median frequency of peripheral Ki67^+^ Treg cells in total T cells at baseline. *P* value was calculated by two-sided log-rank test. **h** Dynamic change of the frequency of Ki67^+^ Tregs in total T cells during treatment compared to baseline levels (in relapse-free group, *n* = 12 at t1−t3 and *n* = 10 at t4; in relapse group, *n* = 12 at t1−t3 and *n* = 8 at t4). *P* values were calculated by two-sided Wilcoxon signed-rank tests. Box plots represent the median (center line), the IQR (box), and the farthest data point within a maximum of 1.5 × IQR (whiskers)

Given the complex composition of immune cell subsets in PBMCs, we employed the machine-learning algorithm CellCnn^17^ to cope with high dimensionality of the data and try to identify the immune features most strongly associated with disease relapse after anti-PD-1 therapy. A CD3^+^CD4^+^CD25^+^FOXP3^+^ Treg-like subpopulation with high expression of Ki67 and low expression of CD45RA at baseline was selected by CellCnn (Fig. 3a and Supplementary Fig. 4a), which resembled an activated and proliferating Treg phenotype.^18^ Back-projection of this CellCnn-selected population to the t-SNE map also showed a notable overlap with the CD4^+^ T cell cluster (94% of the selected cells), especially the CD4^+^ Treg subset (accounting for nearly 56% of the selected cells that were annotated as CD4^+^ T cells, which was more than six times the proportion of Treg cells in CD4^+^ T cells from PBMC) (Figs. 2c, 3b and Supplementary Fig. 4b, c). Patients who developed relapse had a significantly higher frequency of this population at baseline (*P* < 0.001; Fig. 3c). Univariate Cox analyses also indicated the unfavorable prognostic significance of CD4^+^ Treg population at baseline (HR = 4.8, 95% CI: 1.26–18.34; Supplementary Fig. 4d). Furthermore, among functional markers on baseline Tregs, only Ki67 was found to be differently expressed between patients with or without relapse, with the former group exhibiting significantly higher levels (*P* = 0.006; Fig. 3d), consistent with the results of the CellCnn analysis.

We utilized flow cytometry to validate the observations made in the CyTOF analyses. This involved detecting Treg cells in baseline PBMCs 0 patients, with 51 receiving aPD1-CRT therapy and 69 receiving CRT alone. The results confirmed higher frequencies of baseline Treg cells in CD4^+^ T cells and Ki67^+^ Treg cells in Treg cells (gating strategy shown in Supplementary Fig. 5) in patients who eventually developed relapse in the aPD1-CRT arm (*P* = 0.033 and 0.048, respectively; Fig. 3e). Additionally, the frequency of Ki67^+^ Treg in the total CD3^+^ T cell population (Ki67^+^ Treg/T) was higher in patients with relapse compared to those who were relapse-free after aPD1-CRT therapy (*P* = 0.004), but this difference was not observed in patients who received CRT alone (*P* = 0.964; Fig. 3f). Furthermore, a higher level of Ki67^+^ Treg/T was associated with shorter EFS in the aPD1-CRT arm (*P* = 0.008; Supplementary Fig. 6). The link between peripheral Ki67^+^ Treg cells and poor outcomes was also observed in other types of cancer. Analyses of single-cell RNA sequencing (scRNA-seq) data of PBMCs from head and neck squamous cell carcinoma patients who received neoadjuvant ICI therapy^19^ revealed that the frequency of Treg cells expressing MKI67 (gene encoding Ki67) at baseline was notably higher in patients with disease progression (*P* = 0.044; Supplementary Fig. 7).

We next explored the predictive value of Ki67^+^ Treg/T. aPD1-CRT significantly prolonged EFS compared to CRT alone in patients with low Ki67^+^ Treg/T (*P* = 0.045), but the EFS benefit was not observed in those with high Ki67^+^ Treg/T (*P* = 0.487; Fig. 3g). These results suggest that the baseline frequency of peripheral Ki67^+^ Treg cells can predict disease relapse and the treatment efficacy of anti-PD-1 therapy.

### In-depth analyses reveal the potential role of myeloid cells in favoring the generation of Ki67^+^ Treg cells

To gain a comprehensive view of immune interactions, we performed correlation analyses between the frequency of Ki67^+^ Tregs and the frequencies of all identified immune cell subpopulations. We did not observe significant association of the frequency of Ki67^+^ Tregs with lymphocytic subsets (Supplementary Fig. 8). On the other hand, myeloid cells, particularly dendritic cells (DCs), are known to facilitate Treg generation.^20^ Therefore, we analyzed the correlation between the frequency of Ki67^+^ Tregs and that of myeloid cell subsets (including cMo, iMo, ncMo, and DC). The frequency of Ki67^+^ Tregs was positively associated with the frequencies of HLA-DR^+^ and CD86^+^ monocytes and dendritic cells (Supplementary Fig. 9), suggesting their potential roles in the generation of Ki67^+^ Treg cells via antigen presentation and CD28-dependent signaling. Interestingly, we found that the frequency of Ki67^+^ Tregs was also positively associated with the frequencies of PD-L1^+^ monocytes and dendritic cells (Supplementary Fig. 9), which is consistent with previous reports that DCs could induce Treg cells via PD-L1.^21^ These results indicate that myeloid cells might play a crucial role in Treg generation.

### Correlation and association of peripheral and intratumoral Ki67^+^ Treg cells with immunosuppressive tumor microenvironment

Ki67^+^ Treg represents a highly proliferative subset. To gain more insight into the biological function of this subset, we compared the expression of functional markers between Ki67^+^ and Ki67^−^ Tregs (Supplementary Fig. 10). The results demonstrated significantly higher expression of FOXP3, CD38, HLA-DR, and CD39 on Ki67^+^ Tregs, indicating potent immunosuppressive functions of this population.^18,22–24^ Intriguingly, Ki67^+^ Treg also displayed higher expression of PD-1 compared to the Ki67^−^ subset, indicating that anti-PD-1 treatment will be accompanied by further expansion of this population.^25^ Indeed, the frequency of Ki67^+^ Treg in total T cells increased earlier and profounder during treatment in patients who eventually developed relapse (Fig. 3h).

**Fig. 4:**
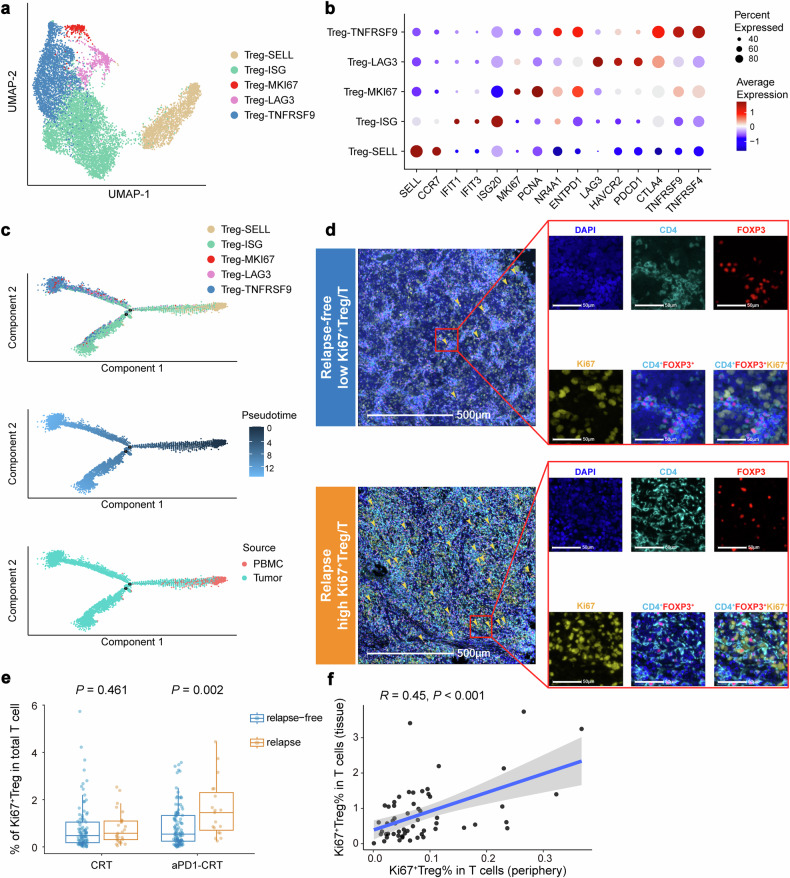
Peripheral Ki67^+^ Treg cells reflect the abundance of their counterparts within the tumor microenvironment. **a** UMAP projection of 10,766 Treg cells colored by clusters. **b** Dot plot showing expression of representative signature genes of the five clusters of Treg cells. Each dot indicates a gene, with color representing the average expression level and size representing the percentage of cells that express the gene. **c** Pseudotime trajectories for Treg cells (Treg-SELL, Treg-ISG, Treg-MKI67, Treg-LAG3, and Treg-TNFRSF9; *n* = 10,766). Each dot represents one single cell, colored according to its cluster label (top), pseudotime score (middle) or sample source (bottom), respectively. **d** Representative mIHC plots showing the existence of CD4^+^FOXP3^+^Ki67^+^ Treg cells (arrow) in tumor tissues from patients with different levels of Ki67^+^ Tregs. Scale bars, 500 and 50 µm for left and right panels, respectively. **e** Box plots showing the frequency of Ki67^+^ Treg cells in total T cells compared between the relapse-free group and the relapse group in the CRT and the aPD1-CRT arms of the CONTINUUM trial, respectively (*n* = 121 and 128, respectively). Box plots represent the median (center line), the IQR (box), and the farthest data point within a maximum of 1.5 × IQR (whiskers). *P* values were calculated using two-sided Wilcoxon tests. **f** Spearman’s correlation between the frequency of intratumoral Ki67^+^ Treg cells and peripheral Ki67^+^ Treg cells (*n* = 58). mIHC multiplex immunohistochemistry

Interestingly, CCR4 and CCR5, receptors for the cytokines CCL22 and CCL5 that are mainly produced by tumor cells, macrophages and DCs, were also highly expressed on Ki67^+^ Tregs (Supplementary Fig. 10), suggesting increased infiltration into the TME.^26^ We further investigated the association between Tregs in the periphery and those in the TME by analyzing matched PBMC and tumor samples from a publicly available scRNA-seq dataset for NPC.^27^ A total of 10,766 Treg cells were identified and clustered into five previously reported Treg subsets,^27–29^ including Treg-SELL, Treg-ISG, Treg-MKI67, Treg-LAG3, and Treg-TNFRSF9 (Fig. 4a). In line with our CyTOF analyses, functional analysis revealed that the Treg-MKI67 cluster, characterized by high expression of proliferative markers (*MKI67*, *PCNA*), also displayed high expression of *NR4A1* (a TCR signaling marker) and some suppressive markers (e.g., *ENTPD1*), but expressed low levels of both naïve markers (*SELL*, *CCR7*) and tissue-resident highly suppressive effector Treg markers (*CTLA4*). This suggests a phenotype of a recently activated Treg subset upon encountering antigens^30^ (Fig. 4b).

The trajectory analyses of the Treg subclusters revealed an inferred developmental track, indicating that Treg cells from the circulation were located at the early stage of differentiation (Fig. 4c). Quantitative analysis of the sample source demonstrated that Treg cells expressing *MKI67* were predominantly located within tumor tissues, accounting for a higher frequency of Tregs in the TME (193/8907, 2.2%) compared to those in the peripheral blood (20/1859, 1.1%). Subsequent mIHC staining of baseline primary tumor samples from 249 patients confirmed the presence of Ki67^+^ Tregs within tumor tissues (Fig. 4d). Additionally, Ki67^+^ Treg/T was significantly higher in patients with relapse compared to relapse-free patients in the aPD1-CRT arm, but this difference was not observed in the CRT arm (*P* = 0.002 and 0.461, respectively; Fig. 4e). Correlation analysis further revealed a significant positive correlation between intratumoral and peripheral Ki67^+^ Tregs (Spearman *R* = 0.45, *P* < 0.001; Fig. 4f), suggesting that circulating Ki67^+^ Treg cells are associated with tumor-infiltrating Treg cells and can reflect the abundance of proliferative Tregs in the TME.

**Fig. 5:**
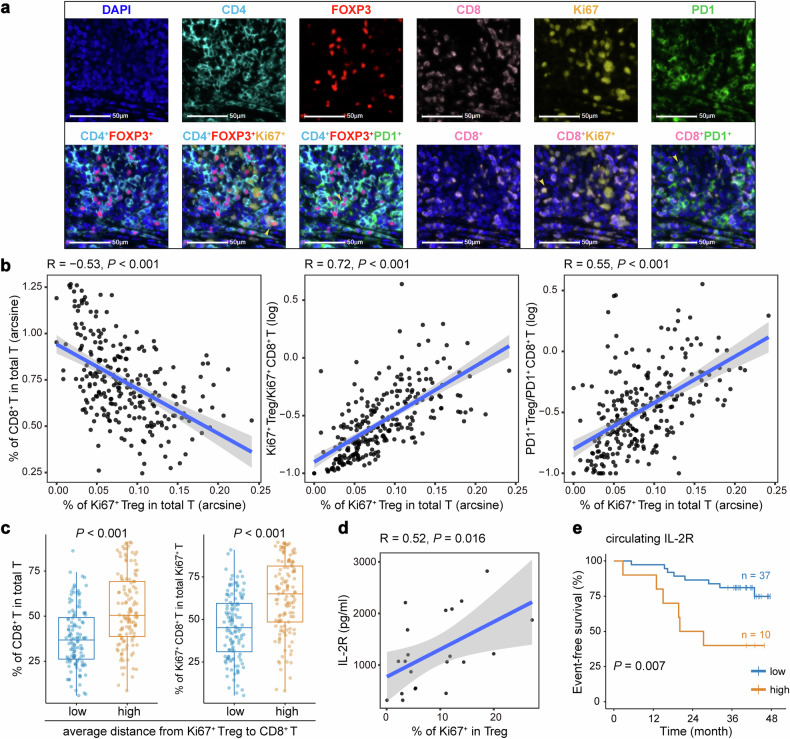
Intratumoral Ki67^+^ Treg cells correlate with an immunosuppressive tumor microenvironment. **a** Representative mIHC plots showing the existence of CD4^+^FOXP3^+^ Treg cells and CD8^+^ T cells expressing Ki67 and PD-1 in the TME (arrow). Scale bars, 50 µm. **b** Spearman’s correlation of the frequency of intratumoral Ki67^+^ Treg cells with the frequency of CD8^+^ T cells, the ratio of Ki67^+^ Tregs to Ki67^+^CD8^+^ T cells, and the ratio of PD-1^+^ Tregs to PD-1^+^CD8^+^ T cells, respectively (*n* = 249). Percentages were arcsine-transformed and ratios were log_10_-transformed. **c** Box plots showing the frequency of CD8^+^ T cells in total T cells and the frequency of Ki67^+^CD8^+^ T cells in total Ki67^+^ T cells compared between groups with a short or long average distance from Ki67^+^ Tregs to CD8^+^ T cells (*n* = 249). Box plots represent the median (center line), the IQR (box), and the farthest data point within a maximum of 1.5 × IQR (whiskers). *P* values were calculated using two-sided Wilcoxon tests. **d** Spearman’s correlation between the frequency of intratumoral Ki67^+^ Treg cells and the concentration of circulating IL-2R at baseline (*n* = 21). **e** Kaplan–Meier curve of the event-free survival of patients with a high or low concentration of circulating IL-2R at baseline. *P* values were generated from log-rank tests. mIHC multiplex immunohistochemistry, TME tumor microenvironment

We proceeded to investigate the effects of the Ki67^+^ Treg population on the TME. Notably, the frequency of Ki67^+^ Treg cells was found to be negatively associated with the frequency of CD8^+^ T cells (Spearman *R* = −0.53, *P* < 0.001) and positively associated with the ratio of Ki67^+^ Tregs to Ki67^+^CD8^+^ T cells, as well as the ratio of PD-1^+^ Tregs to PD-1^+^CD8^+^ T cells (Spearman *R* = 0.72 and 0.55, respectively, both *P* < 0.001; Fig. 5a, b). These associations reflected a balance skewed towards immunosuppression and were linked to worse outcomes with ICI therapy in previous studies.^25,31^ Proximity analyses revealed that the closer adjacency of CD8^+^ T cells to Ki67^+^ Treg cells, indicated by a shorter average distance, correlated with a lower frequency of CD8^+^ T cells and a lower frequency of Ki67^+^CD8^+^ T cells in total Ki67^+^ T cells (both *P* < 0.001; Fig. 5c). This suggests a decrease in both the quantity and proliferation of cytotoxic cells. Furthermore, analysis of circulating proteins associated with immune and inflammatory activity was performed on baseline plasma samples from 47 patients in the aPD1-CRT arm, which revealed a positive correlation between the concentration of soluble IL-2R, an important immunosuppressive factor released by Treg cells,^32^ and the frequency of Ki67^+^ Treg cells in the TME (Spearman *R* = 0.52, *P* = 0.016; Fig. 5d). Interestingly, a higher concentration of circulating IL-2R was associated with significantly inferior EFS after aPD1-CRT (*P* = 0.007; Fig. 5e). Collectively, our observations in both the TME and circulation demonstrate the potential immune suppressive function of Ki67^+^ Treg cells.

### Intratumoral Ki67^+^ Treg cells are predictive of the efficacy of anti-PD-1 therapy

**Fig. 6:**
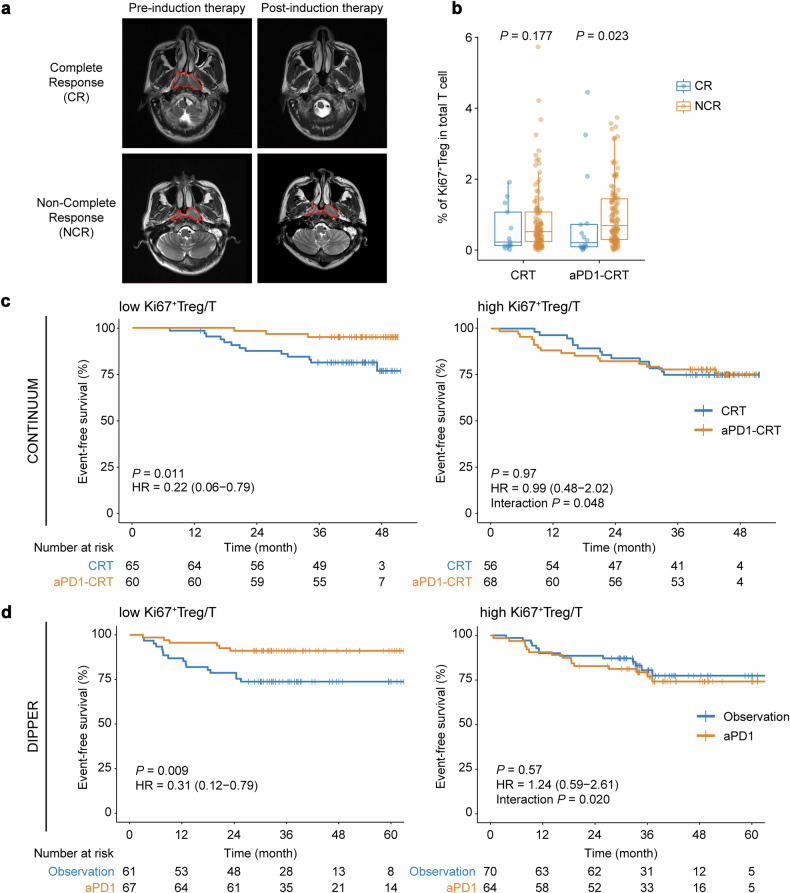
Intratumoral Ki67^+^ Treg cells predict efficacy of anti-PD-1 therapy. **a** Magnetic resonance imaging showing the tumor size in complete responders and non-complete responders before and after induction chemo-immunotherapy, respectively. **b** Box plots showing the ratio of Ki67^+^ Treg to total T cell between CR group and NCR group compared in the CRT arm and in the aPD1-CRT arm, respectively (*n* = 119 and 126, respectively). Box plots represent the median (center line), the IQR (box), and the farthest data point within a maximum of 1.5 × IQR (whiskers). *P* values were calculated using two-sided Wilcoxon tests. **c** Kaplan–Meier curves comparing EFS improvements for anti-PD-1 plus CRT versus CRT alone in patients from the CONTINUUM trial stratified by the median frequency of intratumoral Ki67^+^ Treg cells in total T cells at baseline. **d** Kaplan–Meier curves comparing EFS improvements for anti-PD-1 versus observation in patients from the phase 3 DIPPER trial. The median Ki67^+^ Treg/T in the CONTINUUM trial was used to stratify patients into low/high groups in both CONTINNUM and DIPPER trials. *P* values were generated from log-rank tests, and the HRs were calculated using univariable Cox regression analysis. CR complete response, NCR non-complete response, EFS event-free survival, HR hazard ratio

We then investigated the clinical significance of the intratumoral Ki67^+^ Treg population. Ki67^+^ Treg/T efficiently discriminated complete responders from non-complete responders after aPD1-IC but not IC alone (*P* = 0.023 and 0.177, respectively; Fig. 6a, b). To further assess the predictive value of Ki67^+^ Treg/T in differentiating EFS benefits, we stratified patients into two groups based on median Ki67^+^ Treg/T level (0.0577). Additional aPD1 significantly improved EFS in the Ki67^+^ Treg/T-low group (log-rank *P* = 0.011, HR = 0.22, 95% CI: 0.06–0.79), while EFS was almost the same in both treatment arms in the Ki67^+^ Treg/T-high group (log-rank *P* = 0.97, HR = 0.99, 95% CI: 0.48–2.02; interaction *P* = 0.048; Fig. 6c).

To independently validate our findings in an external cohort, we performed mIHC using 262 FFPE samples from another phase 3 randomized immunotherapy trial (DIPPER trial^11^; NCT03427827). In line with our previous results, Ki67^+^ Treg/T was significantly higher in patients who relapsed compared to non-relapsers in the aPD1 arm, while not in the Observation arm from the DIPPER trial (*P* = 0.016 and 0.888, respectively; Supplementary Fig. 11a). The results also showed a significant inverse correlation between the frequency of Ki67^+^ Tregs and CD8^+^ T cells (Supplementary Fig. 11b). Using the same cut-off value we used in the CONTINUUM cohort (0.0577) to dichotomize patients into Ki67^+^ Treg/T-low or high groups, the survival analysis showed that adding anti-PD-1 significantly prolonged EFS in the Ki67^+^ Treg/T-low group (log-rank *P* = 0.009, HR = 0.31, 95% CI: 0.12–0.79), while not in the Ki67^+^ Treg/T-high group (log-rank *P* = 0.57, HR = 1.24, 95% CI: 0.59–2.61; interaction *P* = 0.020; Fig. 6d), further validating our findings in the CONTINUUM trial cohort.

We also evaluated the prognostic value of Ki67^+^ Treg/T in different treatment arms, and the results from both trials consistently showed that lower Ki67^+^ Treg/T was associated with longer EFS in NPC patients who received aPD1-CRT but not in those who received CRT alone (Supplementary Fig. 12). Therefore, Ki67^+^ Treg cells can also serve as a prognostic factor in patients who received immunotherapy.

To further explore the potential of Ki67^+^ Treg cells as a general predictor of ICI response and benefit in other types of cancer including NSCLC and melanoma, we calculated a Ki67^+^ Treg signature score using bulk RNA sequencing data and classified patients into low and high groups according to the median score. Using datasets from phase 3 randomized controlled trials of anti-PD-L1 immunotherapy in NSCLC,^33^ we found that anti-PD-L1 immunotherapy conferred survival benefits for patients with a low Ki67^+^ Treg score, but not for those with a high score (Supplementary Fig. 13a). For melanoma,^34^ patients with a lower Ki67^+^ Treg score showed better survival after anti-PD-1 immunotherapy (Supplementary Fig. 13b). The above results further demonstrated the generalizability of our findings. Overall, these results demonstrate that a lower frequency of Ki67^+^ Treg cells can predict the benefit of immunotherapy and assist in patient selection.

## Discussion

In this study, we utilized longitudinally collected PBMC samples to perform dynamic immune profiling by CyTOF for biomarker discovery and identified Ki67^+^ Tregs as predictive of immunotherapy benefit. The frequency of this Treg subset was at a significantly higher baseline level and increased during treatment in patients who developed relapse compared to those who did not. Our results also showed a positive correlation between peripheral Ki67^+^ Tregs and intratumoral Ki67^+^ Tregs, both of which demonstrated consistent prognostic and predictive value.

Tregs play a critical role in immune homeostasis and are mainly comprised of two subsets with distinct phenotypes and functions: CD45RA^+^FOXP3^lo^ resting Treg cells (rTreg) and CD45RA^–^FOXP3^hi^ activated Treg cells (aTreg). When stimulated, rTreg cells express Ki67, proliferate and convert into aTreg cells to exert their regulatory functions.^18^ The Ki67^+^ Treg subset identified in this study displayed significantly higher expression of FOXP3, CD38, HLA-DR, and CD39, resembling activated and proliferating Treg cells that possess high immune suppressive functions and may contribute to inducing a hypo-activated state of anti-tumor immunity. In patients with relapse after immunotherapy, higher levels of Treg and naïve cells, and lower levels of terminal effector cells were observed in circulation. We also observed an inverse relationship between the frequency of intratumoral Ki67^+^ Treg cells and CD8^+^ T cells, indicating an immunosuppressive TME associated with Ki67^+^ Treg cells. Interestingly, it is known that anti-cytotoxic T-lymphocyte-associated protein 4 (CTLA-4) can inhibit the suppressive function of Tregs mediated by CTLA-4, as well as deplete Tregs via Fc-mediated antibody-dependent cellular cytotoxicity and antibody-dependent cellular phagocytosis.^26^ Hence, the combination of anti-PD-1 and anti-CTLA-4 may potentially counteract the adverse impact of Tregs and overcome resistance to anti-PD-1 therapy in patients with elevated levels of Ki67^+^ Tregs. Our group is conducting a phase 3 trial testing the efficacy of cadonilimab, a bi-specific antibody targeting PD-1/CTLA-4, in locoregionally advanced NPC, which may provide further evidence for this strategy (NCT05587374).

Previous researches have reported elevated Treg levels in the peripheral blood of NPC patients compared to healthy individuals, and an increase in tumor-infiltrating Tregs, suggesting a connection between these two compartments.^35,36^ In this study, we revealed a positive correlation between the frequency of peripheral Ki67^+^ Tregs and the frequency of intratumoral Ki67^+^ Treg population. CyTOF analysis showed that compared with the Ki67^−^ counterparts, Ki67^+^ Tregs expressed higher levels of CCR4 and CCR5, receptors for the chemotactic factors CCL22 and CCL5 that are mainly produced by tumor cells, macrophages and DCs in the TME. Pseudotime analysis also revealed a developmental trajectory from blood to tumor tissue, suggesting that activated Ki67^+^ Treg cells in the periphery may migrate into tissues to form subpopulations with heightened functionality and interact with other components in the TME. The frequency of Ki67^+^ Treg cells showed a significantly positive correlation with myeloid cells, but not with lymphocytes, indicating a possible role for myeloid cells in the formation of Ki67^+^ Treg cells. Nevertheless, the exact site of activation remains unclear. Previous reports have indicated that Treg cells expressing Ki67, NR4A1 (an early marker of TCR signaling), and immune regulatory markers such as CD39 represent a subset of activated cells that have recently encountered antigens, either during priming in the lymph node or upon reactivation in local tissue.^30^ Further investigation is needed to understand the generation of the Ki67^+^ Treg population we identified, and to determine whether these cells are peripherally activated or locally activated prior to recirculation. This will facilitate the development of treatment strategies targeting this cell subpopulation.

Acknowledging the limitations of our present study is crucial. Constrained by the scarcity of published randomized ICI trials with publicly available biomarker datasets, we were only able to validate the predictive value of Ki67^+^ Tregs in one randomized phase 3 trial in NSCLC. To further confirm the predictive value of Ki67^+^ Tregs in various malignancies, it is imperative to conduct future prospectively designed biomarker analyses embedded in randomized trials, similar to the current study.

Through the prospectively designed biomarker analyses of this randomized, controlled, exploratory clinical biomarker dataset, we have furnished evidence demonstrating the clinical significance of Ki67^+^ Treg cells as a determinant of survival benefit from anti-PD-1 treatment. These findings have the potential to shape future individualized treatment strategies and inform the development of novel combination treatments for patients with NPC and other tumor types. Moreover, this study underscores the unique and indispensable role of randomized controlled trials in identifying robust predictive biomarkers, thereby advancing the realization of precision medicine.

## Materials and methods

### Study design and sample collection

The multicenter randomized phase 3 CONTINUUM trial (NCT03700476) evaluated the efficacy of aPD1-CRT versus CRT alone in patients with primary locoregionally advanced NPC.^10^ The trial enrolled patients in nine sites across China between December 2018 and March 2020 and involved the prospective collection of longitudinal blood samples and baseline formalin-fixed paraffin-embedded (FFPE) primary tumor samples from patients in both arms (Fig. 1a). Blood samples were obtained at four time points: before any therapy (baseline; t1), after anti-PD-1 ± induction chemotherapy (aPD1 ± IC; t2), after anti-PD-1 ± concurrent chemoradiotherapy (aPD1 ± CCRT; t3), and at the end of adjuvant anti-PD-1 therapy (aPD1 alone; t4). Blood was collected and processed within 1 h of phlebotomy. Whole blood was centrifuged at 1000 × *g* for 10 min at room temperature. Plasma was carefully collected, aliquoted, and stored at −80 °C until use. PBMCs were isolated by density gradient centrifugation in Lymphocytes Separation Medium (TBD, Tianjin, China) for 20 min at 400 × *g*. PBMCs were then washed with normal saline solution and cryopreserved at −80 °C in Serum-Free Cell Freezing Medium (New Cell & Molecular Biotech, Suzhou, China) until use.

In the discovery cohort, CyTOF was performed on PBMC samples from patients with or without posttreatment relapse after aPD1-CRT treatment, who were well matched based on age, sex, stage, and PD-L1 expression (Supplementary Table 1). All remaining available baseline PBMC samples from patients receiving aPD1-CRT or CRT alone in the CONTINUUM trial were used for flow cytometry. Additionally, all available pretreatment FFPE primary tumor samples from patients in the CONTINUUM trial underwent mIHC staining to validate the predictive value of the Ki67^+^ Treg subset that was identified (Fig. 1b).

The multicenter randomized phase 3 DIPPER trial^11^ (NCT03427827) evaluated the efficacy of adjuvant aPD1 versus observation after curative chemoradiation in patients with primary locoregionally advanced NPC. The trial enrolled patients in 11 sites across China between August 2018 and November 2021. All available pretreatment FFPE primary tumor samples from patients in the DIPPER trial underwent mIHC staining for the validation of the predictive value of the Ki67^+^ Treg subset (Fig. 1b). The protocols for both trials were approved by the institutional ethics review board at each participating center. Written informed consent was obtained from all patients.

### Mass cytometry (CyTOF)

All CyTOF antibodies were pre-conjugated from Fluidigm (Supplementary Table 6). Cryopreserved cell suspensions were thawed at 37 °C. Then, 1~3 × 10^6^ PBMCs in single-cell suspension were stained with Cell-ID Cisplatin (Fluidigm) at room temperature for 2 min to identify viable cells. Samples were labeled and pooled for staining using the Cell-ID 20-Plex Pd Barcoding Kit (Fluidigm). Cells were subsequently blocked with Human TruStain FcX (BioLegend) at room temperature for 10 min to minimize non-specific background staining, followed by incubation with surface antibodies at room temperature for 30 min. After incubation with Nuclear Antigen Staining Buffer (Fluidigm), cells were incubated with nuclear antibodies in Nuclear Antigen Staining Perm (Fluidigm) at room temperature for 30 min. Cells were then stained with Cell-ID Intercalator-Ir (Fluidigm) and stored at 4 °C until acquisition. After resuspending cells in Cell Acquisition Solution (Fluidigm), sample acquisition was conducted using the Helios CyTOF system (Fluidigm).

### CyTOF data analysis

We first applied cyCombine^37^ to perform batch correction and data integration. CyTOF data were analyzed with R using modified functions from cytofWorkflow.^38^ The FlowSOM algorithm combined with consensus clustering was used for identification of clusters and the t-distributed stochastic neighbor embedding (t-SNE) algorithm was used for visualization. Heatmap of canonical markers of each subpopulation was shown and used for manual annotation of the clusters. Then, major cell types were extracted for subclustering analyses using the same procedure. CD8^+^ T cells were clustered based on CD45RO, CD45RA, CCR7, CD28 and CD69, and were annotated as CD45RA^+^CCR7^+^ naïve cells (CD8^+^Tnaïve), CD45RA^−^CCR7^+^ central memory cells (CD8^+^Tcm), CD45RA^−^CCR7^−^ effector memory cells (CD8^+^Tem) and CD45RA^+^CCR7^−^ terminally differentiated effector memory cells (CD8^+^Temra).^15,16^ CD4^+^ T cells were clustered based on CD45RO, CD45RA, CCR7, CD28, CD127, CD69 and CD25, and were annotated as CD45RA^+^CCR7^+^ naïve cells (CD4^+^Tnaïve), CD45RA^−^CCR7^+^ central memory cells (CD4^+^Tcm), CD45RA^−^CCR7^−^ effector memory cells (CD4^+^Tem), CD45RA^+^CCR7^−^ terminally differentiated effector memory cells (CD4^+^Temra) and CD25^+^CD127^−^ regulatory T cells (CD4^+^Treg). CD19^+^ B cells were clustered based on CD19, CD21, CD24, CD27 and CD38, and were annotated as CD27^−^CD38^−^ naïve cells (Bnaïve), CD27^+^CD38^−^ memory cells (Bmem), CD27^+^CD38^+^ plasmablast-like cells (Plasmablast), and CD24^hi^CD27^+^ regulatory B cell-like cluster (Breg-like).^16,39^ CD33^+^ myeloid cells were clustered based on CD14, CD16, CD11b, CD11c and HLA-DR, and were annotated as CD14^+^CD16^−^ classical monocytes (cMo), CD14^+^CD16^+^ intermediate monocytes (iMo), CD14^−^CD16^+^ non-classical monocytes (ncMo) and CD11c^+^HLA-DR^+^CD14^−^CD16^−^ conventional dendritic cells (cDCs).^40^ NK cells were clustered based on CD56, CD16, CCR7 and CD69, and were annotated as CD56^bright^ NK and CD56^dim^ NK.^41^ Once all clusters were annotated, cluster frequencies were calculated as the percentage of parental cell population.

### CellCnn analysis

CellCnn^17^ is a supervised representation learning model that is designed to detect rare cell subpopulations associated with disease status or survival information from high-dimensional single-cell data. The model predicts disease phenotypes based on relative frequencies of automatically selected cell subpopulation via a convolutional filter. We used all baseline cells and all markers as inputs and ran CellCnn with default parameters. We then performed two-sided Wilcoxon tests to test whether the identified cell population is significantly over-represented in patients in the relapse group.

### Flow cytometry

Baseline PBMCs were analyzed using flow cytometry. Briefly, PBMCs were treated with Human TruStain FcX (BioLegend) to prevent non-specific staining, and then incubated with surface antibodies for 30 min at 4 °C. For nuclear staining, cells were fixed and permeabilized using FOXP3 fix/perm buffer set (eBioscience) according to the manufacturer’s instructions. Fixable Viability Dye eFluor 520 was used to identify living cells. Samples were analyzed on a CytoFLEX LX instrument (Beckman Coulter) and the results were processed using FlowJo software (TreeStar). Details of the antibodies used can be found in Supplementary Table 7.

### Single-cell RNA-seq data analysis

We analyzed public scRNA-seq data with matched samples from tumor and PBMC using Seurat v.4.3.0.^42^ Doublets were identified using DoubletFinder^43^ and were removed. After normalization, we performed batch correction and data integration by the reciprocal PCA (RPCA). We then applied principal component analysis (PCA) to reduce dimension and visualized data with uniform manifold approximation and projection (UMAP) using the top 2000 highly variable genes and the top 30 principal components. The *FindClusters* function was used for cell clustering with a resolution parameter set to 0.6, resulting in 26 clusters. All identified clusters were annotated as one of the major cell types based on the expression of canonical marker genes. We then selected CD4^+^ Treg cells and re-ran the aforementioned analyses, resulting in five clusters. Representative genes expression among these clusters was examined and displayed with the *DotPlot* function.

### Pseudotime trajectories analysis

Pseudotime analysis for scRNA-seq data was performed using Monocle2 (v2.26.0)^44^ to infer developmental trajectories of Treg cell subsets. Marker genes across all clusters were identified and those with adjusted *P* values <0.001 were regarded as significant and used to order cells. Dimension reduction was performed with the *reduceDimension* function using the *DDRTree* algorithm, and cell pseudotime trajectories were visualized with the *plot_cell_trajectory* function.

### Multiplex immunohistochemistry (mIHC)

The mIHC was performed using the PANO 6-plex IHC kit (Panovue, Beijing, China) as reported previously.^45,46^ Briefly, FFPE tissue samples were sliced into 4-μm-thick sections and mounted onto positive charged slides, heated at 60°C for 1 h, followed by deparaffinization and rehydration. After treatment with blocking buffer (Beyotime, Shanghai, China) for 30 min, the sections were serially stained with different primary antibodies (Supplementary Table 8), followed by horseradish peroxidase-conjugated secondary antibodies treatment and tyramide signal amplification (TSA). The slides were microwave heat-treated for antigen retrieval and antibody stripping after each round of TSA operation. Nuclei were counterstained with 4′,6-diamidino-2-phenylindole (DAPI) after all sequential staining steps had been done. Slides were then sealed using the Antifade Mounting Medium (Beyotime, Shanghai, China).

### Image acquisition and analysis

Images were scanned by the Vectra Polaris imaging system (Akoya Biosciences) and analyzed using the HALO software (Indica Labs, NM, USA) as described previously.^47,48^ The neural network Nuclei Seg (Plugin) FL v1.0.0 (Indica labs) algorithm was trained for robust detection of individual cells based on nuclear DAPI staining. Phenotype annotation was performed manually by a scientist determining thresholds for marker positivity for each sample. Cell types were subsequently defined according to the combination of positive markers. Once cell types were assigned, the percentage out of the parental population was calculated for each sample. Spatial analysis between Ki67^+^ Treg cells and CD8^+^ T cells was performed using the HALO Proximity Analysis algorithm with cell object XY coordinates data. Average distance between the two cell types per sample was extracted from the summary results.

### Signature score for Ki67^+^ Treg cells

Publicly available bulk RNA-seq data from two immunotherapy cohorts were retrieved, normalized, and log-transformed. To characterize Ki67^+^ Treg cells in the TME from transcriptomic data, marker genes for Ki67^+^ Treg cluster were obtained from a previous publication^27^ to calculate a signature score using the “zscore” method of R package GSVA.^49^

### Multiplex immune assay for cytokines and chemokines

A total of 65 cytokines, chemokines, and growth factors/regulators in the plasma were simultaneously measured by the multiplexed Luminex xMAP assay (Immune Monitoring 65-Plex Human ProcartaPlex™ Panel, ThermoFisher Scientific, see Supplementary Table 9). All assays were conducted as per manufacturer’s protocols.

### Statistical analysis

The primary endpoint was EFS, and the secondary endpoints were DMFS and OS. We calculated EFS from randomization to disease relapse (distant metastasis or locoregional recurrence) or death from any cause; DMFS to the first distant metastasis; and OS to death due to any cause. Treatment responses after induction chemo-immunotherapy were evaluated using the laryngopharyngeal endoscopy and magnetic resonance imaging according to the Response Evaluation Criteria in Solid Tumors version 1.1.^50^

All analyses were performed using R software (v4.1.1). We used the median value in each dataset to dichotomize patients into high and low Ki67^+^ Treg groups. The specific reference values are summarized in Supplementary Table 10. For each group, unstratified hazard ratios and corresponding 95% CIs were calculated using an unstratified Cox model with study group as the only covariate. Cox models were utilized to generate interaction *P* values by incorporating an interaction term between treatment assignment and the biomarker-defined subgroups. Kaplan–Meier plots were created, and log-rank *P* values were determined using the survminer (v0.4.9) and survival packages (v3.2-11). All *P* values were two-sided, and the level of significance was set at 0.05 for all statistical tests unless otherwise noted.

## Supplementary information

Supplementary Materials

## Source data

Source Data

## Data Availability

Publicly available bulk RNA-seq and scRNA-seq datasets analyzed in this study were retrieved from the Gene Expression Omnibus (GEO) database (www.ncbi.nlm.nih.gov/geo/) with the accession numbers GSE91061, GSE200996, and GSE162025. Bulk RNA-seq data of the phase 3 randomized OAK trial were retrieved from the European Genome-phenome Archive (EGAS00001005013). All the key raw research data were uploaded onto the Research Data Deposit public platform (www.researchdata.org.cn) with the approval number RDDB2024118962, and could be obtained upon reasonable request. Source data are provided with this paper.
