# Supplementary material for: Systemic longitudinal immune profiling identifies proliferating Treg cells as predictors of immunotherapy benefit: biomarker analysis from the phase 3 CONTINUUM and DIPPER trials
Source: Signal Transduct Target Ther. 2024 Oct 23;9:285. doi: 10.1038/s41392-024-01988-w (PMC11496634; doi:10.1038/s41392-024-01988-w)
Supplement: Supplementary file 1 — Supplementary Materials [file 41392_2024_1988_MOESM1_ESM.pdf]

Supplementary Materials for

Systemic longitudinal immune profiling identifies proliferating Treg cells as predictors of immunotherapy benefit: biomarker analysis from the phase 3 CONTINUUM and DIPPER trials

Sai-Wei Huang, Wei Jiang, Sha Xu, Yuan Zhang, Juan Du, Ya-Qin Wang, Kun-Yu Yang, Ning Zhang, Fang Liu, Guo-Rong Zou, Feng Jin, Hai-Jun Wu, Yang-Ying Zhou, Xiao-Dong Zhu, Nian-Yong Chen, Cheng Xu, Han Qiao, Na Liu, Ying Sun, Jun Ma, Ye-Lin Liang, Xu Liu

Correspondence to: liuxu@sysucc.org.cn; liangyl@sysucc.org.cn; majun2@mail.sysu.edu.cn.

This PDF file includes:

Tables S1 to S10
Figures S1 to S13

Supplementary Table 1. Clinicopathological characteristics of 12 pairs of LA-NPC patients with or without relapse after anti-PD-1 treatment in the CONTINUUM trial.

	Relapse-free, N (%) n = 12	Relapse, N (%) n = 12	*P* value
**Age**			> 0.999
≤45	4 (33.3)	4 (33.3)	
>45	8 (66.7)	8 (66.7)	
**Sex**			.
male	12 (100.0)	12 (100.0)	
**T category**			> 0.999
T2	0 (0.0)	1 (8.3)	
T3	5 (41.7)	4 (33.3)	
T4	7 (58.3)	7 (58.3)	
**N category**			> 0.999
N1	3 (25.0)	2 (16.7)	
N2	2 (16.7)	3 (25.0)	
N3	7 (58.3)	7 (58.3)	
**TNM stage**			.
IV	12 (100.0)	12 (100.0)	
**EBV DNA**			0.714
<4000	4 (33.3)	2 (16.7)	
≥4000	7 (58.3)	8 (66.7)	
unknown	1 (8.3)	2 (16.7)	
**PD-L1**			0.856
negative	2 (16.7)	2 (16.7)	
positive	7 (58.3)	5 (41.7)	
unknown	3 (25.0)	5 (41.7)	

The *P* values were determined using the two-tailed χ^2 tests or Fisher's exact tests.

Abbreviations: TNM, tumor-node-metastasis; EBV, Epstein-Barr virus.

Supplementary Table 2. Clinicopathological characteristics of LA-NPC patients from the CONTINUUM trial included in the current biomarker study.

	ITT (n=425)	FCM (n=120)		mIHC (n=249)	
	N (%)	N (%)	*P* value vs. ITT	N (%)	*P* value vs. ITT
**Age**			0.147		0.783
≤45	204 (48.0)	48 (40.0)		116 (46.6)	
>45	221 (52.0)	72 (60.0)		133 (53.4)	
**Sex**			0.434		0.759
female	110 (25.9)	36 (30.0)		61 (24.5)	
male	315 (74.1)	84 (70.0)		188 (75.5)	
**ECOG PS**			0.189		0.821
0	405 (95.3)	118 (98.3)		239 (96.0)	
1	20 (4.7)	2 (1.7)		10 (4.0)	
**T category**			0.986		0.856
T1	11 (2.6)	3 (2.5)		4 (1.6)	
T2	47 (11.1)	14 (11.7)		29 (11.6)	
T3	165 (38.8)	48 (40.0)		99 (39.8)	
T4	202 (47.5)	55 (45.8)		117 (47.0)	
**N category**			0.587		0.724
N1	91 (21.4)	25 (20.8)		51 (20.5)	
N2	195 (45.9)	61 (50.8)		109 (43.8)	
N3	139 (32.7)	34 (28.3)		89 (35.7)	
**TNM stage**			0.403		0.998
III	126 (29.6)	41 (34.2)		73 (29.3)	
IV	299 (70.4)	79 (65.8)		176 (70.7)	
**EBV DNA**			0.423		0.385
<4000	276 (69.3)	87 (73.7)		155 (65.7)	
≥4000	122 (30.7)	31 (26.3)		81 (34.3)	
**PD-L1**			0.302		> 0.999
negative	56 (21.8)	20 (28.6)		52 (21.8)	
positive	201 (78.2)	50 (71.4)		187 (78.2)	

The *P* values were determined using the two-tailed χ^2 tests or Fisher's exact tests.

Abbreviations: ITT, intention-to-treat; ECOG PS, the Eastern Cooperative Oncology Group Performance Status; TNM, tumor-node-metastasis; EBV, Epstein-Barr virus; FCM, flow cytometry; mIHC, multiplex immunohistochemistry.

Supplementary Table 3. Clinicopathological characteristics of LA-NPC patients from the DIPPER trial included in the current biomarker study.

	ITT (n = 450)<br>N (%)	mIHC (n = 262)<br>N (%)	*P* value
**Age**			0.656
≤45	221 (49.1%)	134 (51.1%)	
>45	229 (50.9%)	128 (48.9%)	
**Sex**			0.420
female	110 (24.4%)	72 (27.5%)	
male	340 (75.6%)	190 (72.5%)	
**ECOG PS**			0.272
0	103 (22.9%)	50 (19.1%)	
1	347 (77.1%)	212 (80.9%)	
**T category**			0.778
T1	19 (4.22%)	13 (4.96%)	
T2	42 (9.33%)	28 (10.7%)	
T3	182 (40.4%)	110 (42.0%)	
T4	207 (46.0%)	111 (42.4%)	
**N category**			0.805
N1	88 (19.6%)	46 (17.6%)	
N2	220 (48.9%)	131 (50.0%)	
N3	142 (31.6%)	85 (32.4%)	
**TNM stage**			0.651
III	139 (30.9%)	86 (32.8%)	
IV	311 (69.1%)	176 (67.2%)	
**EBV DNA**			0.797
<4000	336 (74.7%)	190 (72.5%)	
≥4000	105 (23.3%)	67 (25.6%)	
unknown	9 (2.00%)	5 (1.91%)	
**PD-L1**			>0.999
negative	40 (15.1%)	40 (15.3%)	
positive	225 (84.9%)	222 (84.7%)	

The *P* values were determined using the two-tailed χ^2 tests or Fisher's exact tests.
Abbreviations: CRT, chemoradiotherapy; aPD1, anti-PD-1; ECOG PS, the Eastern Cooperative Oncology Group Performance Status; TNM, tumor-node-metastasis; EBV, Epstein-Barr virus.

Supplementary Table 4. Clinicopathological characteristics of LA-NPC patients from the phase 3 CONTINUUM trial in this study.

	FCM (n=120)			mIHC (n=249)		
	CRT (n=69)	aPD1-CRT (n=51)	*P* value	CRT (n=121)	aPD1-CRT (n=128)	*P* value
**Age**			0.274			0.588
≤45	31 (44.9%)	17 (33.3%)		59 (48.8%)	57 (44.5%)	
>45	38 (55.1%)	34 (66.7%)		62 (51.2%)	71 (55.5%)	
**Sex**			0.468			0.736
female	23 (33.3%)	13 (25.5%)		28 (23.1%)	33 (25.8%)	
male	46 (66.7%)	38 (74.5%)		93 (76.9%)	95 (74.2%)	
**ECOG PS**			>0.999			0.336
0	68 (98.6%)	50 (98.0%)		118	121 (94.5%)	
1	1 (1.45%)	1 (1.96%)		3 (2.48%)	7 (5.47%)	
**T category**			>0.999			0.585
T1	2 (2.90%)	1 (1.96%)		2 (1.65%)	2 (1.56%)	
T2	8 (11.6%)	6 (11.8%)		15 (12.4%)	14 (10.9%)	
T3	27 (39.1%)	21 (41.2%)		43 (35.5%)	56 (43.8%)	
T4	32 (46.4%)	23 (45.1%)		61 (50.4%)	56 (43.8%)	
**N category**			0.271			0.868
N1	15 (21.7%)	10 (19.6%)		26 (21.5%)	25 (19.5%)	
N2	31 (44.9%)	30 (58.8%)		51 (42.1%)	58 (45.3%)	
N3	23 (33.3%)	11 (21.6%)		44 (36.4%)	45 (35.2%)	
**TNM stage**			0.676			0.166
III	22 (31.9%)	19 (37.3%)		30 (24.8%)	43 (33.6%)	
IV	47 (68.1%)	32 (62.7%)		91 (75.2%)	85 (66.4%)	
**EBV DNA**			0.790			0.857
<4000	52 (75.4%)	35 (71.4%)		78 (66.7%)	77 (64.7%)	
≥4000	17 (24.6%)	14 (28.6%)		39 (33.3%)	42 (35.3%)	
**PD-L1**			>0.999			0.478
negative	11 (29.7%)	9 (27.3%)		28 (24.1%)	24 (19.5%)	
positive	26 (70.3%)	24 (72.7%)		88 (75.9%)	99 (80.5%)	

The *P* values were determined using the two-tailed χ^2 tests or Fisher's exact tests.

Abbreviations: CRT, chemoradiotherapy; aPD1, anti-PD-1; ECOG PS, the Eastern Cooperative Oncology Group Performance Status; TNM, tumor-node-metastasis; EBV, Epstein-Barr virus; FCM, flow cytometry; mIHC, multiplex immunohistochemistry.

Supplementary Table 5. Clinicopathological characteristics of LA-NPC patients from the phase 3 DIPPER trial in this study.

	Observation (n=131)	aPD1 (n=131)	*P* value
**Age**			0.902
≤45	66 (50.4%)	68 (51.9%)	
>45	65 (49.6%)	63 (48.1%)	
**Sex**			0.489
female	39 (29.8%)	33 (25.2%)	
male	92 (70.2%)	98 (74.8%)	
**ECOG PS**			0.875
0	24 (18.3%)	26 (19.8%)	
1	107 (81.7%)	105 (80.2%)	
**T category**			0.105
T1	6 (4.58%)	7 (5.34%)	
T2	20 (15.3%)	8 (6.11%)	
T3	50 (38.2%)	60 (45.8%)	
T4	55 (42.0%)	56 (42.7%)	
**N category**			0.228
N1	25 (19.1%)	21 (16.0%)	
N2	70 (53.4%)	61 (46.6%)	
N3	36 (27.5%)	49 (37.4%)	
**TNM stage**			0.693
III	45 (34.4%)	41 (31.3%)	
IV	86 (65.6%)	90 (68.7%)	
**EBV DNA**			0.462
<4000	95 (72.5%)	95 (72.5%)	
≥4000	32 (24.4%)	35 (26.7%)	
unknown	4 (3.1%)	1 (0.8%)	
**PD-L1**			0.864
negative	19 (14.5%)	21 (16.0%)	
positive	112 (85.5%)	110 (84.0%)	

The *P* values were determined using the two-tailed χ^2 tests or Fisher's exact tests.

Abbreviations: CRT, chemoradiotherapy; aPD1, anti-PD-1; ECOG PS, the Eastern Cooperative Oncology Group Performance Status; TNM, tumor-node-metastasis; EBV, Epstein-Barr virus.

Supplementary Table 6. Mass cytometry panel antibody information.

Antigen	Clone	Label	Source	Cat#	Dilution
CD45	HI30	89Y	Fluidigm	3089003B	1:200
CD3	UCHT1	141Pr	Fluidigm	3141019B	1:200
CD40	5C3	142Nd	Fluidigm	3142010B	1:100
CD127	A019D5	143Nd	Fluidigm	3143012B	1:100
CD69	FN50	144Nd	Fluidigm	3144018B	1:100
CD4	RPAT4	145Nd	Fluidigm	3145001B	1:200
CD8	RPAT8	146Nd	Fluidigm	3146001B	1:200
CD11c	Bu15	147Sm	Fluidigm	3147008B	1:200
CD16	3G8	148Nd	Fluidigm	3148004B	1:200
CD25	2A3	149Sm	Fluidigm	3149010B	1:100
CD86	IT2.2	150Nd	Fluidigm	3150020B	1:100
CD14	M5E2	151Eu	Fluidigm	3151009B	1:200
CD21	BL13	152Sm	Fluidigm	3152010B	1:100
CCR2	K036C2	153Eu	Fluidigm	3153023B	1:100
TIM3	F382E2	154Sm	Fluidigm	3154010B	1:100
CD56/NCAM	B159	155Gd	Fluidigm	3155008B	1:100
CD183/CXCR3	G025H7	156Gd	Fluidigm	3156004B	1:100
CD194/CCR4	L291H4	158Gd	Fluidigm	3158032A	1:100
CD197/CCR7	G043H7	159Tb	Fluidigm	3159003A	1:100
CD39	A1	160Gd	Fluidigm	3160004B	1:100
Ki-67	B56	161Dy	Fluidigm	3161007B	1:100
FOXP3	PCH101	162Dy	Fluidigm	3162011A	1:100
CD33	WM53	163Dy	Fluidigm	3163023B	1:200
CD45RO	UCHL1	164Dy	Fluidigm	3164007B	1:100
CD19	HIB19	165Ho	Fluidigm	3165025B	1:200
CD24	ML5	166Er	Fluidigm	3166007B	1:100
CD27	O323	167Er	Fluidigm	3167002B	1:100
CD206/MMR	152	168Er	Fluidigm	3168008B	1:100
CD274/PD-L1	MIH1	169Tm	Fluidigm	3169029B	1:100
CD45RA	HI100	170Er	Fluidigm	3170010B	1:100
CD195/CCR5	NP6G4	171Yb	Fluidigm	3171017A	1:100
CD38	HIT2	172Yb	Fluidigm	3172007B	1:100
CD137/4-1BB	4B4-1	173Yb	Fluidigm	3173015B	1:100
HLA-DR	L243	174Yb	Fluidigm	3174001B	1:100
CD279/PD-1	EH12.2H7	175Lu	Fluidigm	3175008B	1:100
CD196/CCR6	G034E3	176Yb	Fluidigm	3176022A	1:100
CD11b	ICRF44	209Bi	Fluidigm	3209003B	1:100

Supplementary Table 7. Flow cytometry panel antibody information.

Marker	Clone	Fluorophore	Source	Dilution
CD3	SK7	APC	Biolegend	1:200
CD4	RPA-T4	PE/Cyanine7	Biolegend	1:200
CD25	BC96	Brilliant Violet 421	Biolegend	1:200
FOXP3	150D	PE	Biolegend	1:100
Ki-67	Ki-67	Alexa Fluor 700	Biolegend	1:100

Supplementary Table 8. Multiplex immunohistochemistry (mIHC) panel antibody information.

Marker	Antibody clone	Source	Dilution	Detection dye (cycle)
PD-1	D4W2J	CST	1:500	520 (1)
Ki67	MIB1	ZsBio	ready to use	570 (2)
CD4	UMAB64	ZsBio	ready to use	480 (3)
FOXP3	D2W8E	CST	1:200	650 (4)
CD8	SP16	ZsBio	1:100	780 (5)

Supplementary Table 9. Cytokines, chemokines, and growth factors/regulators in the Immune Monitoring 65-Plex Human ProcartaPlex™ Panel.

APRIL	GRO-α	IL-2	IL-8	MIP-1β
BAFF	HGF	IL-20	IL-9	MIP-3α
BLC	IFN-α	IL-21	IP-10	MMP-1
CD30	IFN-γ	IL-22	I-TAC	NGF-β
CD40L	IL-10	IL-23	LIF	SCF
ENA-78	IL-12p70	IL-27	MCP-1	SDF-1α
Eotaxin	IL-13	IL-2R	MCP-2	TNF-α
Eotaxin-2	IL-15	IL-3	MCP-3	TNF-β
Eotaxin-3	IL-16	IL-31	M-CSF	TNF-R2
FGF-2	IL-17A	IL-4	MDC	TRAIL
Fractalkine	IL-18	IL-5	MIF	TSLP
G-CSF	IL-1α	IL-6	MIG	TWEAK
GM-CSF	IL-1β	IL-7	MIP-1α	VEGF-A

Supplementary Table 10. Cutoff values for Ki67$^+$ Tregs used in all cohorts and predictive/prognostic value.

Cohort	Cutoff value	Hazard ratio and log-rank *P* value (ICI vs. Control)		Figure No.
		Low Ki67$^+$ Treg group	High Ki67$^+$ Treg group	
CONTINUUM PBMC	Median (0.063)	0.15 (0.02−1.26) $P = 0.045$	1.45 (0.51−4.13) $P = 0.487$	Fig. 4g
CONTINUUM FFPE	Median (0.0577)	0.22 (0.06−0.79) $P = 0.011$	0.99 (0.48−2.02) $P = 0.97$	Fig. 6c
DIPPER FFPE	49th percentile (0.0577, the same value as the CONTINUUM FFPE cohort)	0.35 (0.15−0.86) $P = 0.016$	1.19 (0.56−2.52) $P = 0.657$	Fig. 6d
OAK	Median (0.676)	0.74 (0.57−0.96) $P = 0.0223$	0.95 (0.75−1.21) $P = 0.700$	Fig. S13
*Riaz *et al.*	Median (1.165)	High vs low: 2.10 (1.05−4.18) $P = 0.032$		Fig. S13

Supplementary Figure 1

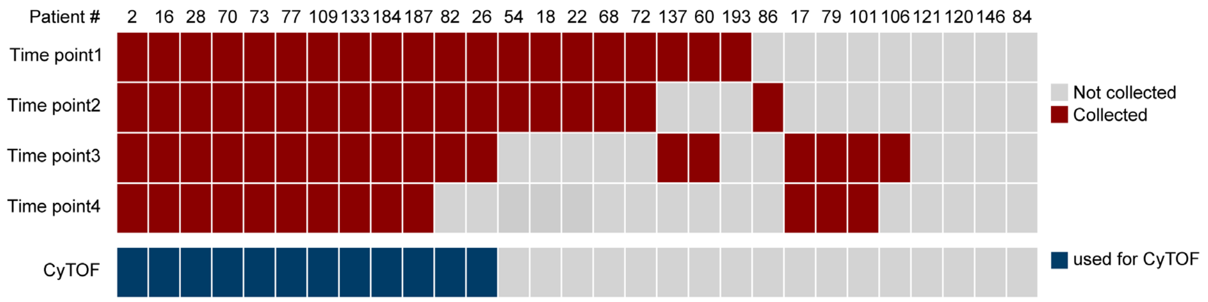

Supplementary Figure 1. PBMC Sample collection from the 29 patients who developed relapse in the aPD1-CRT arm of the CONTINUUM trial.

CyTOF, mass cytometry.

Supplementary Figure 2

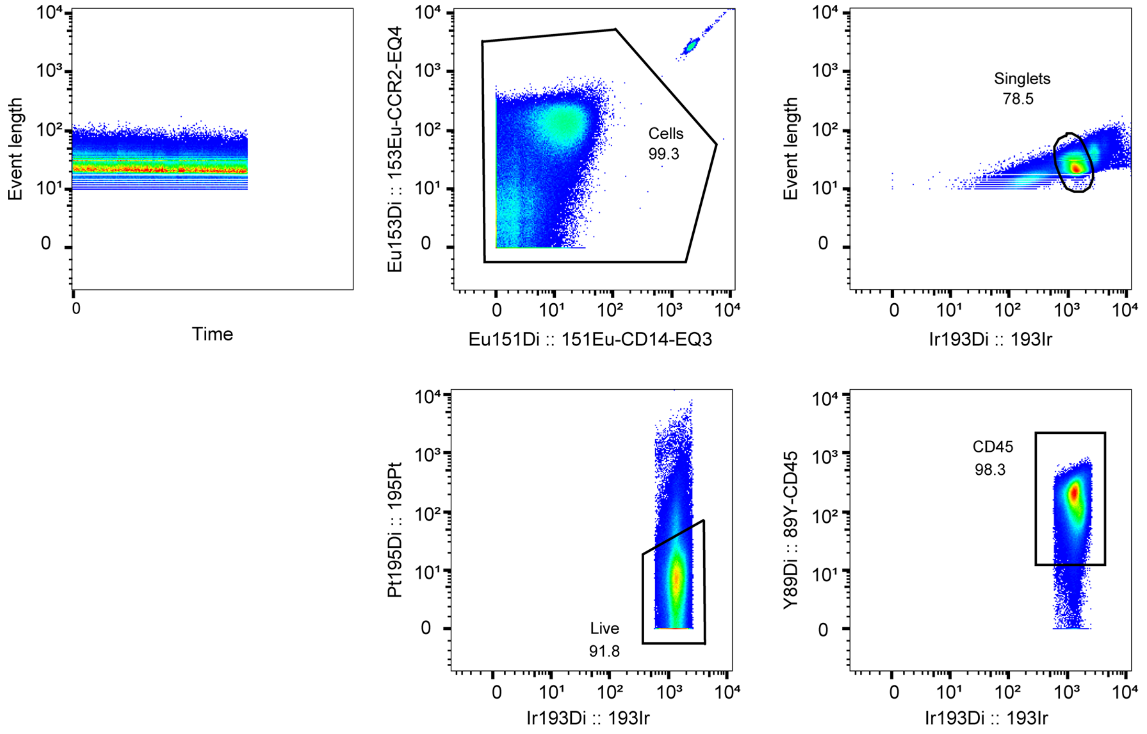

Supplementary Figure 2. CyTOF gating strategy.

Representative flow plots are shown for gating single live CD45$^+$ immune cells for downstream analyses.

Supplementary Figure 3

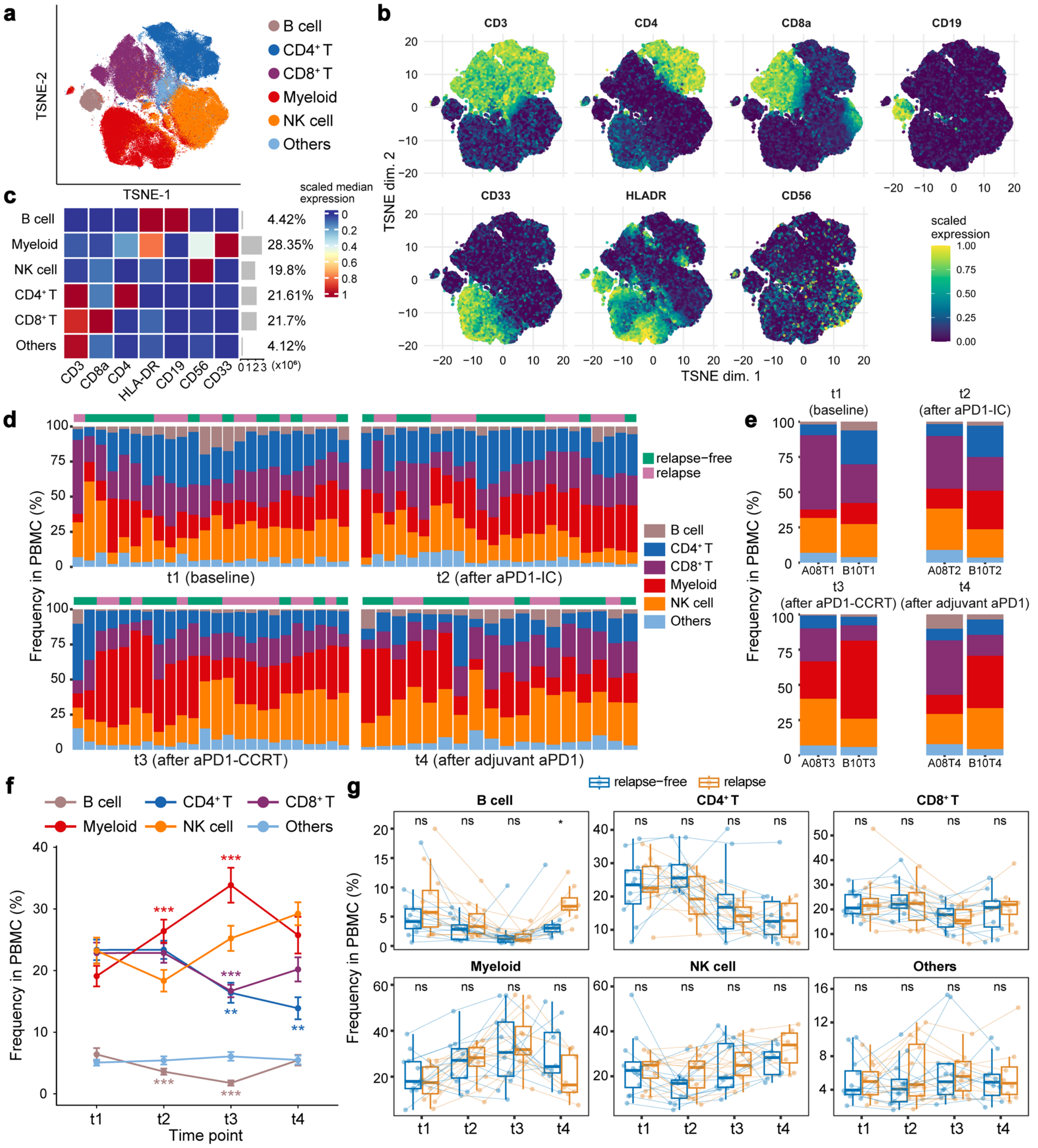

Supplementary Figure 3. Different patterns of major immune cell populations before and during ICI treatment analyzed using CyTOF.

(a) T-SNE plot showing major immune cell types in PBMCs (n = 10,487,969). **(b)** T-SNE plots showing robust expression of major immune canonical markers within the expected immune subsets. **(c)** Heatmap (left) showing expression of canonical markers of each major cell type and histogram (right) showing their composition in PBMCs. **(d)** Phenotypic composition of the major cell types constituting the PBMC cell population shown per sample. Bars at the top of the

plots represent individual samples from relapse-free group (green) and relapse group (pink). **(e)** Stacked bar chart showing the heterogeneous composition of the major cell types in PBMC samples from two patients (A08 & B10). **(f)** Line chart showing the dynamic changes in each major PBMC cell population compared to its baseline level during treatment. *P* values were calculated using Wilcoxon signed-rank tests. **(g)** Box plots showing frequencies of each major cell type in PBMCs between the relapse-free group and the relapse group at each time point of blood collection. For comparisons at each time point, n = 24 for t1−t3 and n = 18 for t4. Box plots represent the median (center line), the IQR (box), and the farthest data point within a maximum of 1.5 × IQR (whiskers). *P* values were calculated using two-sided Wilcoxon tests. *$P < 0.05$, **$P < 0.01$, ***$P < 0.001$; ns, not significant. PBMCs, peripheral blood mononuclear cells.

Supplementary Figure 4

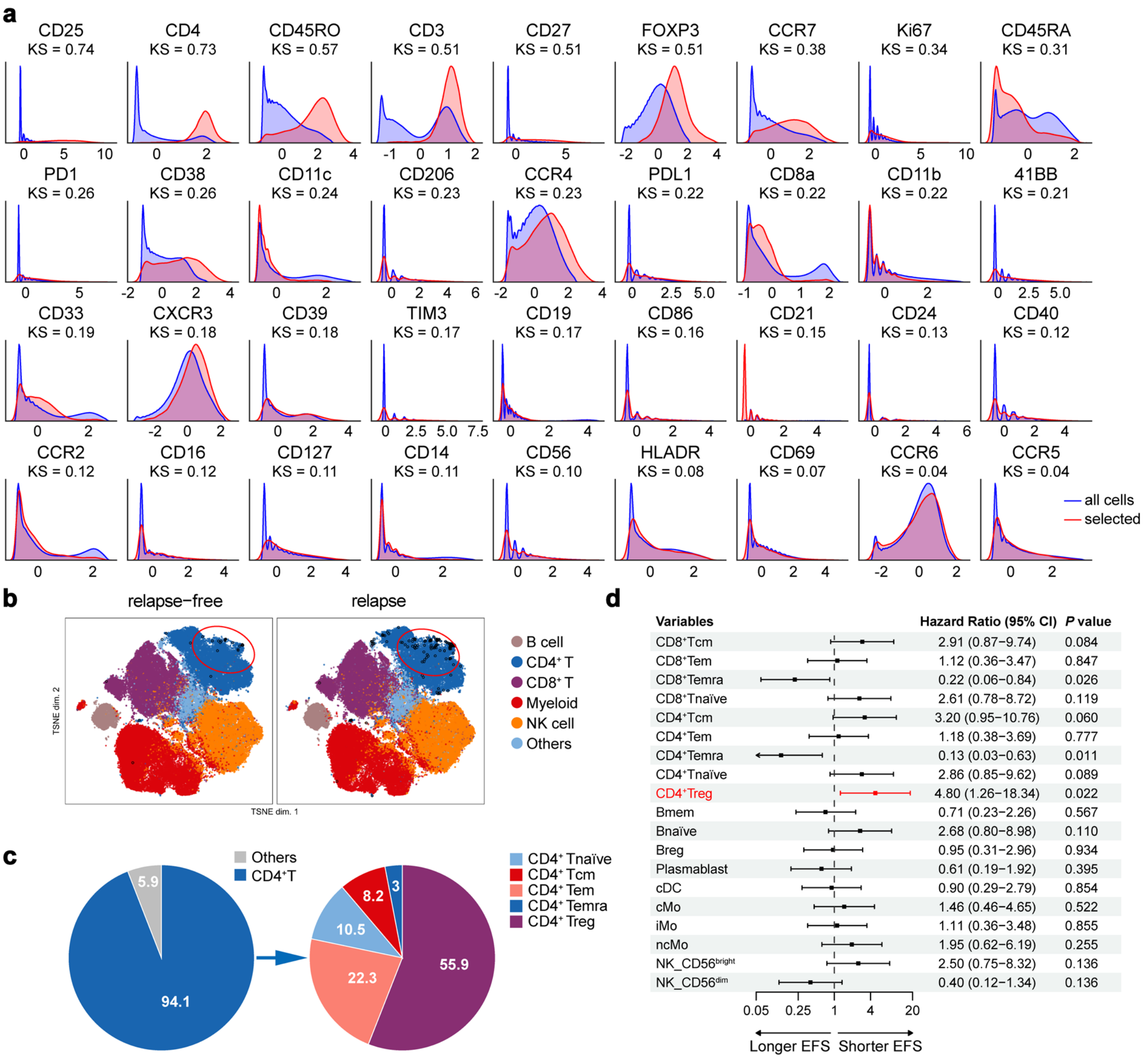

Supplementary Figure 4. CellCnn analysis identified a relapse-associated Treg subset.

(a) Histograms of all measured marker abundances for the whole cell population and the cell subset selected by CellCnn. **(b)** Back projection of cells selected by CellCnn into t-SNE plots corresponding to Figure 2A. Single cells selected by CellCnn are marked with black circles and the populations are indicated with red ellipse, exhibiting a higher frequency in the relapse group. **(c)** Pie charts showing the cell composition in the CellCnn-selected cells based on previous cell annotations. Tnaïve, naïve T cell. Tcm, central memory T cell. Tem, effector memory T cell. Temra, terminally differentiated effector memory T cell. Treg, regulatory T cell. **(d)** Forest plot showing the results from univariate Cox analyses evaluating the prognostic value of the frequencies of all immune cell subpopulations at baseline. EFS, event-free survival. CI, confidence interval.

Supplementary Figure 5

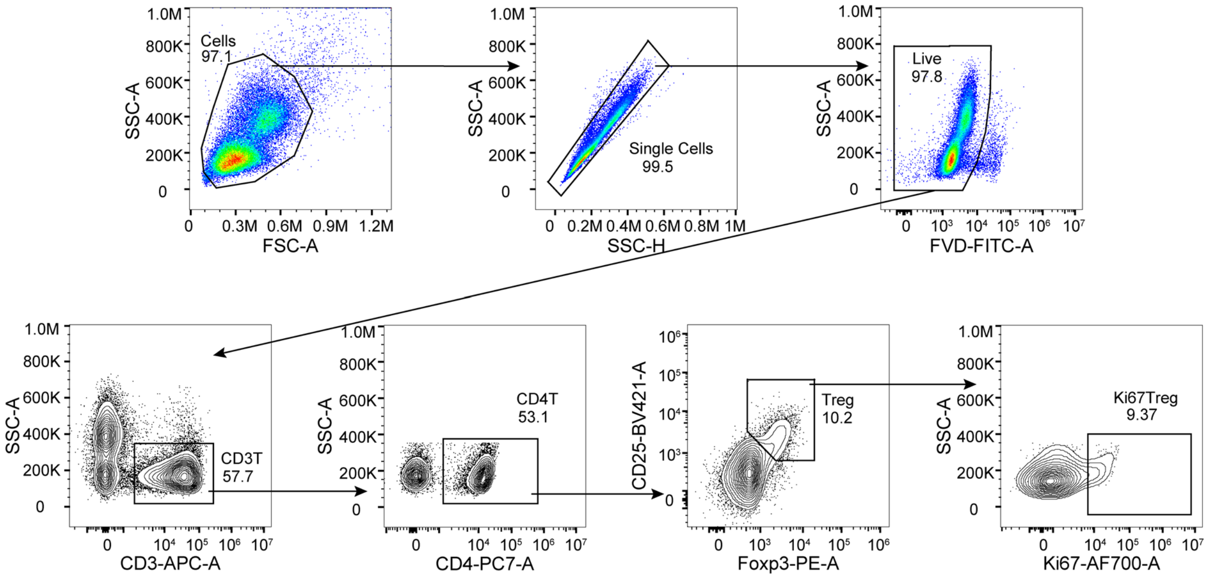

Supplementary Figure 5. Flow cytometry analysis of peripheral Ki67$^+$ Treg cells.

All available baseline PBMC samples from the CONTINUUM trial were stained for CD3, CD4, CD25, FOXP3, and Ki67, acquired and analyzed using the above gating strategy.

Supplementary Figure 6

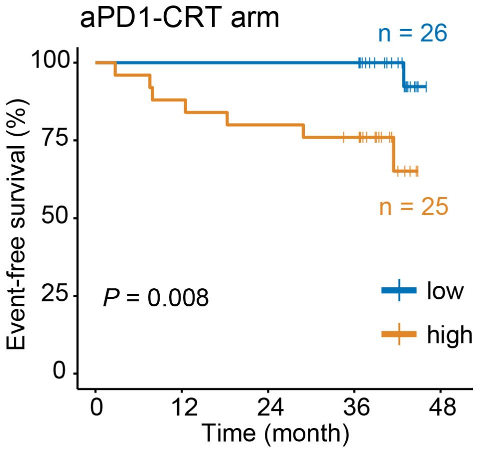

Supplementary Figure 6. Prognostic value of the frequency of Ki67$^+$ Tregs in total T cells in PBMC.

Kaplan-Meier curves of the event-free survival of patients stratified by the median frequency of Ki67$^+$ Tregs in total T cells in PBMC at baseline in the aPD1-CRT arm of the CONTINUUM trial. *P* value was calculated by two-sided log-rank test.

Supplementary Figure 7

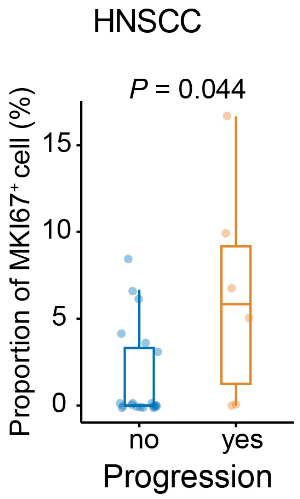

Supplementary Figure 7. Higher frequency of Ki67$^+$ Tregs in HNSCC patients with progression compared to those without progression after immunotherapy.

Box plots showing the frequency of Ki67$^+$ Treg cells in Treg cells compared between head and neck squamous cell carcinoma (HNSCC) patients with or without progression after immunotherapy using data from a single-cell RNA sequencing dataset (GSE200996, n = 26). Box plots represent the median (center line), the IQR (box), and the farthest data point within a maximum of 1.5 × IQR (whiskers). *P* values were calculated by two-sided Wilcoxon signed-rank tests.

Supplementary Figure 8

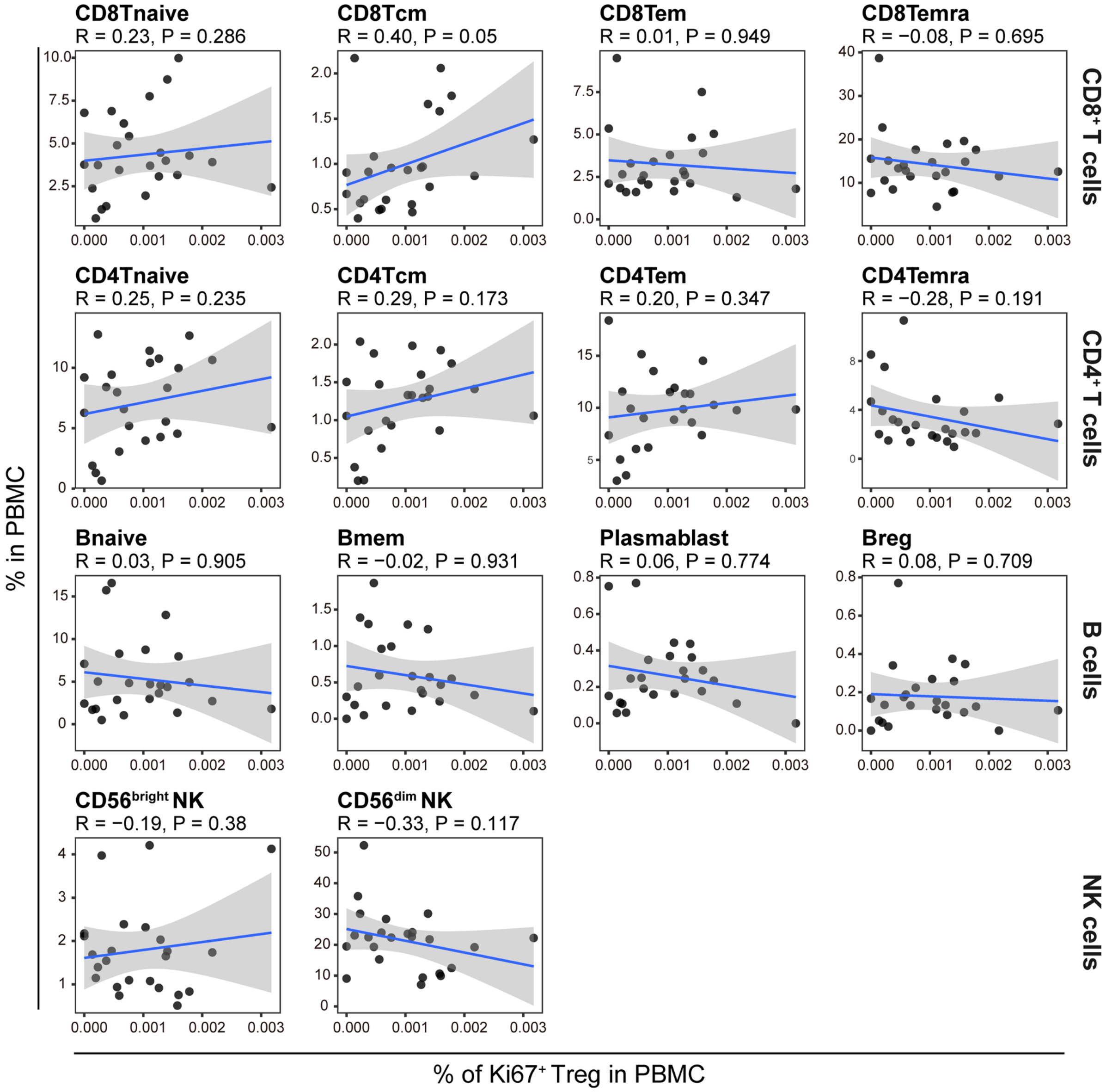

Supplementary Figure 8. Correlation of the frequency of Ki67^{+} Treg cells with the frequencies of lymphocytic subsets in PBMC (n = 24).

Spearman's correlation of the frequency of Ki67^{+} Treg cells with the frequencies of CD8^{+} T cell subsets (CD8^{+} Tnaïve, CD8^{+} Tcm, CD8^{+} Tem, CD8^{+} Temra), CD4^{+} T cell subsets (CD4^{+} Tnaïve, CD4^{+} Tcm, CD4^{+} Tem, CD4^{+} Temra), B cells subsets (Bnaïve, Bmem, Plasmablast, Breg), and NK cell subsets (CD56^{bright} NK, CD56^{dim} NK) in PBMC. Tnaïve, naïve T cell. Tcm, central memory T cell. Tem, effector memory T cell. Temra, terminally differentiated effector memory T cell. Treg, regulatory T cell. Bnaïve, naïve B cell. Bmem, memory B cell. Breg, regulatory B cell. NK, natural killer cell.

Supplementary Figure 9

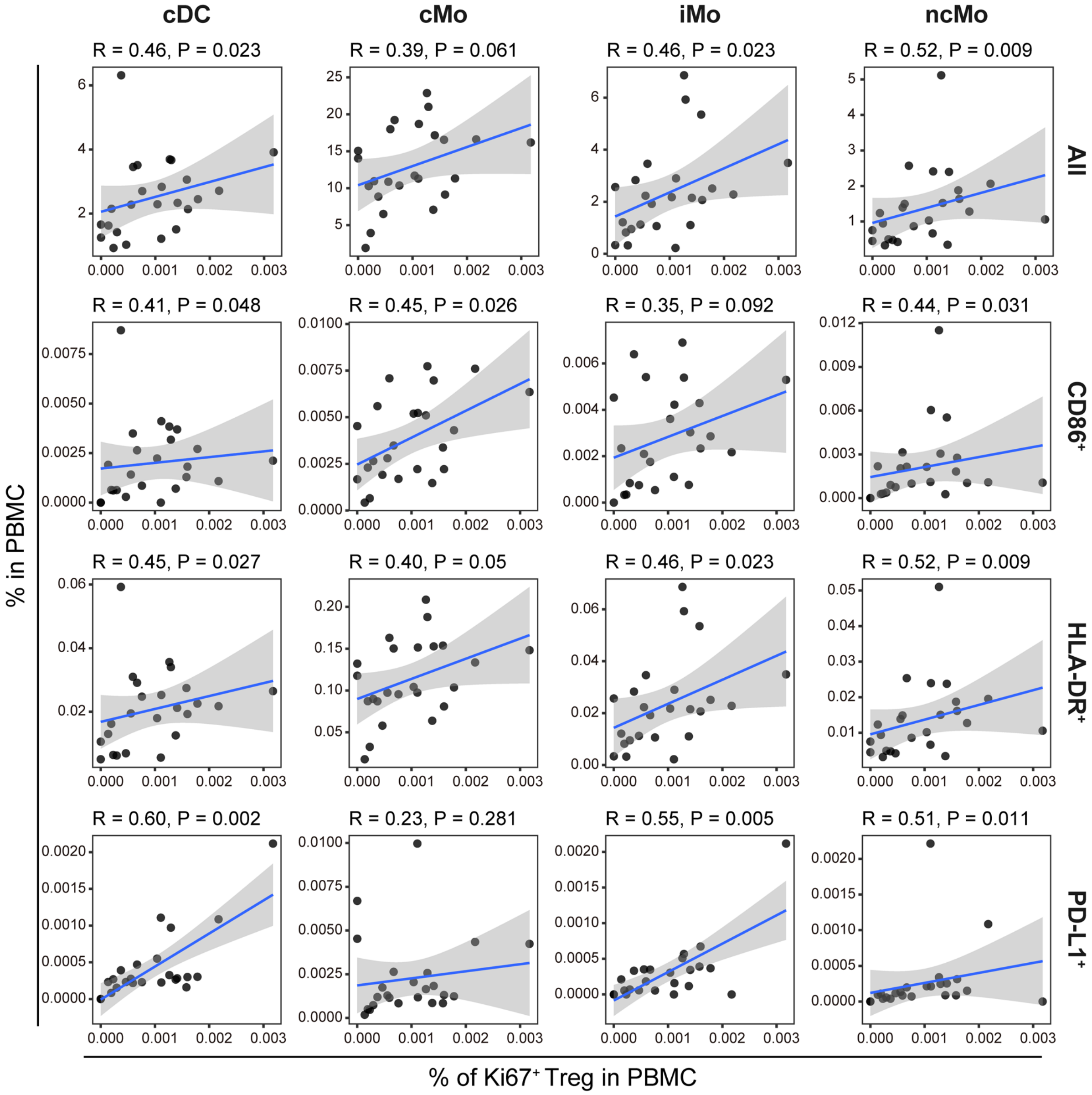

Supplementary Figure 9. Correlation of the frequency of Ki67$^+$ Treg cells with the frequencies of myeloid cell subsets and CD86$^+$, HLA-DR$^+$ or PD-L1$^+$ myeloid cell subsets in PBMC (n = 24).

Spearman's correlation of the frequency of Ki67$^+$ Treg with the frequencies of myeloid cell subsets (cDC, cMo, iMo, and ncMo), and CD86$^+$, HLA-DR$^+$ or PD-L1$^+$ myeloid cell subsets, respectively. cMo, classical monocyte. iMo, intermediate monocyte. ncMo, non-classical monocyte. cDC, conventional dendritic cell.

Supplementary Figure 10

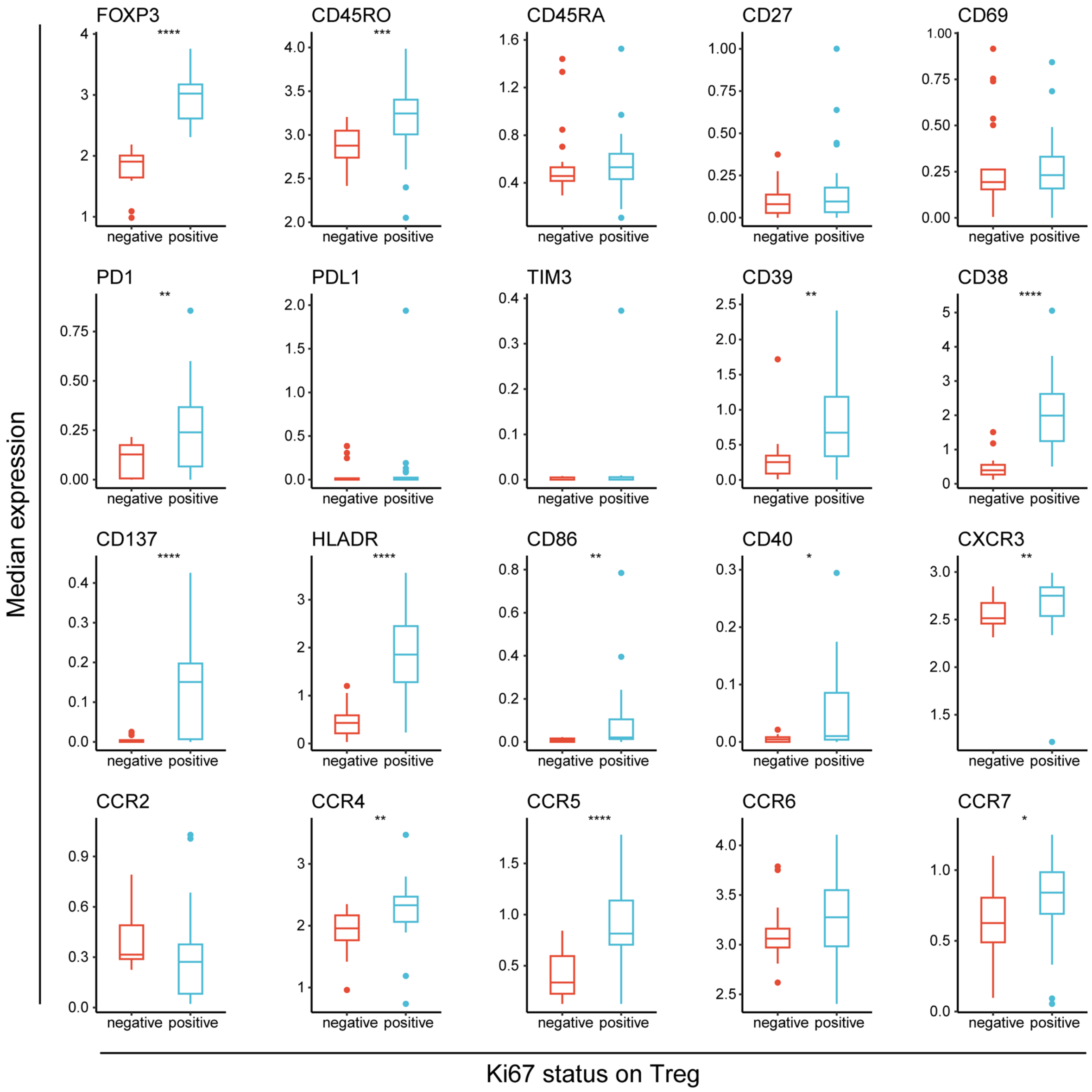

Supplementary Figure 10. Comparisons of the expression of functional markers between Ki67^{+} and Ki67^{-} Tregs in PBMC at baseline (n = 24).

Box plots represent the median (center line), the IQR (box), and the farthest data point within a maximum of 1.5 × IQR (whiskers). *P* values were calculated by two-sided Wilcoxon rank-sum tests and were labelled if significant. $*P < 0.05$, $**P < 0.01$, $***P < 0.001$.

Supplementary Figure 11

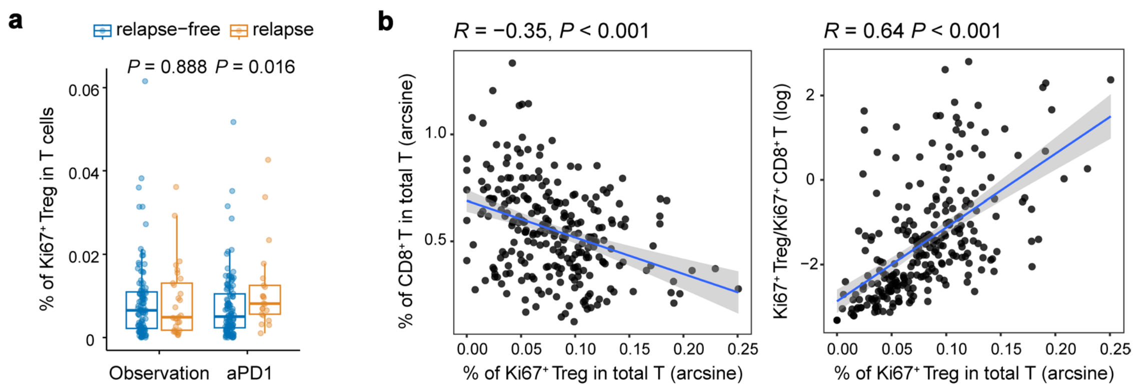

Supplementary Figure 11. Validation of a higher frequency of Ki67$^+$ Tregs in patients who developed relapse in the aPD1 arm and its association with an immunosuppressive TME using data from phase 3 DIPPER trial.

(**a**) Box plots showing the frequency of Ki67$^+$ Treg cells in total T cells between the relapse-free group and the relapse group compared in the Observation arm and in the aPD1 arm, respectively (n = 131 and 131, respectively). Box plots represent the median (center line), the IQR (box), and the farthest data point within a maximum of 1.5 × IQR (whiskers). *P* values were calculated using two-sided Wilcoxon tests. (**b**) Spearman's correlation of the frequency of Ki67$^+$ Treg in total T cells with the frequency of CD8$^+$ T in total T cells and the ratio of Ki67$^+$ Tregs to Ki67$^+$ CD8$^+$ T cells in the DIPPER trial cohort (n = 262). Percentages were arcsine-transformed and ratios were log2-transformed.

Supplementary Figure 12

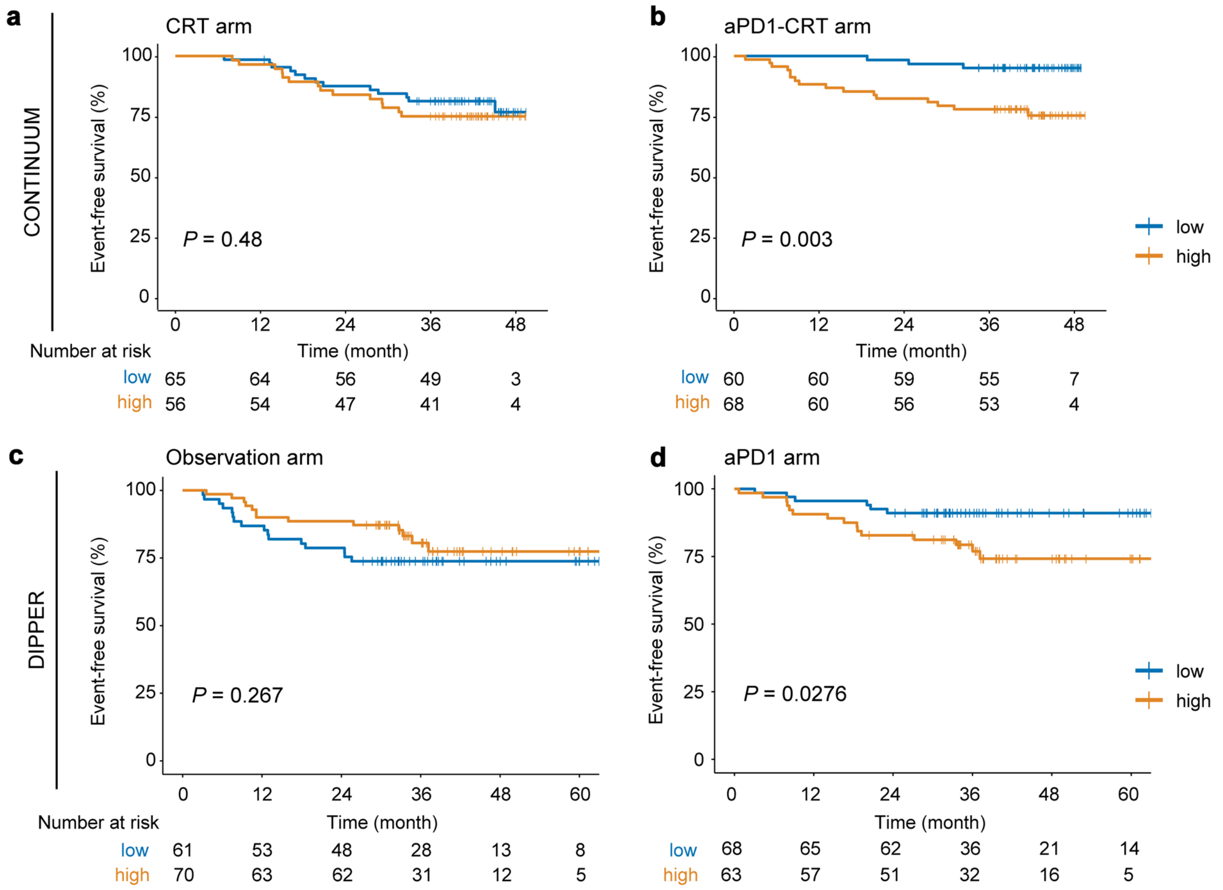

Supplementary Figure 12. Prognostic value of intratumoral Ki67$^+$ Treg/T.

Kaplan-Meier curves of the event-free survival of patients in the CRT arm or the aPD1-CRT arm stratified by intratumoral Ki67$^+$ Treg/T at baseline using data from CONTINUUM **(a, b)** and DIPPER trial **(c, d)**. The median Ki67$^+$ Treg/T in the CONTINUUM trial was used to stratify patients into low/high groups in both trials. Log-rank test was used for analysis.

Supplementary Figure 13

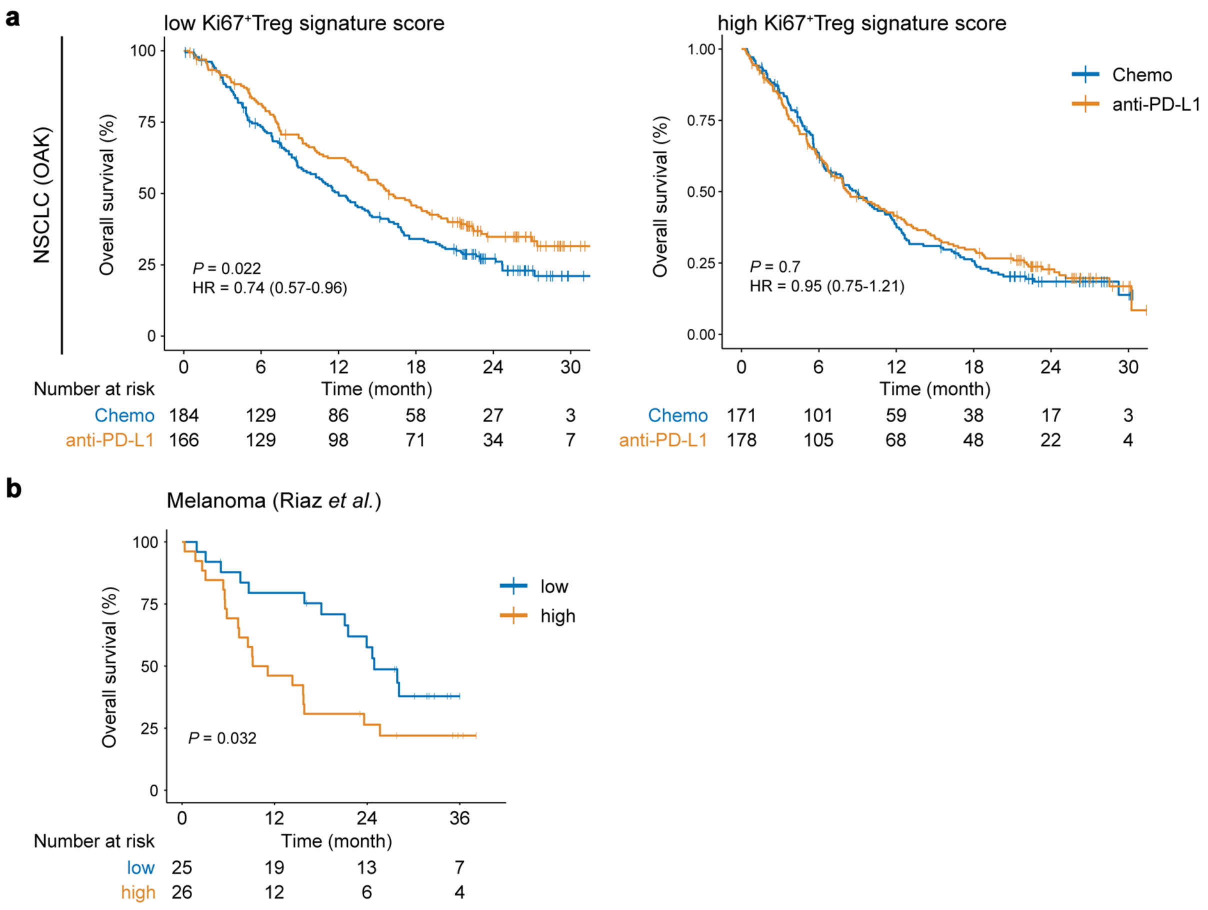

Supplementary Figure 13. Validation of the predictive/prognostic value of intratumoral Ki67^{+} Treg cells using publicly available datasets.

Kaplan-Meier curves of the overall survival of patients from OAK (NSCLC; **a**) and Riaz *et al*. melanoma cohorts **(b)** stratified by the median of Ki67^{+} Treg signature scores. Log-rank test was used for analysis.
